# Microfluidic study of retention and elimination of abnormal red blood cells by human spleen with implications for sickle cell disease

**DOI:** 10.1073/pnas.2217607120

**Published:** 2023-02-02

**Authors:** Yuhao Qiang, Abdoulaye Sissoko, Zixiang L. Liu, Ting Dong, Fuyin Zheng, Fang Kong, John M. Higgins, George E. Karniadakis, Pierre A. Buffet, Subra Suresh, Ming Dao

**Affiliations:** ^a^Department of Materials Science and Engineering, Massachusetts Institute of Technology, Cambridge, MA 02139; ^b^Université Paris Cité, INSERM, Biologie Intégrée du Globule Rouge, 75015 Paris, France; ^c^Université des Antilles, Biologie Intégrée du Globule Rouge, 75015 Paris, France; ^d^Laboratoire d'Excellence du Globule Rouge, 75015 Paris, France; ^e^Division of Applied Mathematics, Brown University, Providence, RI 02912; ^f^Koch Institute for Integrative Cancer Research, Massachusetts Institute of Technology, Cambridge, MA 02139; ^g^School of Biological Sciences, Nanyang Technological University, 639798 Singapore, Singapore; ^h^Massachusetts General Hospital, Harvard Medical School, Boston, MA 02114; ^i^Nanyang Technological University, 639798 Singapore, Singapore

**Keywords:** hypoxia, acute splenic sequestration crisis, splenic retention, erythrophagocytosis, organ-on-a-chip

## Abstract

The spleen clears altered red blood cells (RBCs) from circulation, contributing to the balance between RBC formation (erythropoiesis) and removal. The splenic RBC retention and elimination occur predominantly in open circulation where RBCs flow through macrophages and inter-endothelial slits (IESs). The mechanisms underlying and interconnecting these processes significantly impact clinical outcomes. In sickle cell disease (SCD), blockage of intrasplenic sickled RBCs is observed in infants splenectomized due to acute splenic sequestration crisis (ASSC). This life-threatening RBC pooling and organ swelling event is plausibly triggered or enhanced by intra-tissular hypoxia. We present an oxygen-mediated spleen-on-a-chip platform for in vitro investigations of the homeostatic balance in the spleen. To demonstrate and validate the benefits of this general microfluidic platform, we focus on SCD and study the effects of hypoxia on splenic RBC retention and elimination. We observe that RBC retention by IESs and RBC–macrophage adhesion are faster in blood samples from SCD patients than those from healthy subjects. This difference is markedly exacerbated under hypoxia. Moreover, the sickled RBCs under hypoxia show distinctly different phagocytosis processes from those non-sickled RBCs under hypoxia or normoxia. We find that reoxygenation significantly alleviates RBC retention at IESs, and leads to rapid unsickling and fragmentation of the ingested sickled RBCs inside macrophages. These results provide unique mechanistic insights into how the spleen maintains its homeostatic balance between splenic RBC retention and elimination, and shed light on how disruptions in this balance could lead to anemia, splenomegaly, and ASSC in SCD and possible clinical manifestations in other hematologic diseases.

## Significance

The human spleen modulates the retention and elimination of abnormal red blood cells (RBCs) through inter-endothelial slits and erythrophagocytosis, thereby maintaining blood homeostasis. This balance can be severely disrupted by the increased number of aberrant RBCs under certain pathological conditions, such as in sickle cell disease (SCD). Hypoxia in the spleen can lead to severe consequences, as indicated by various degrees of sickling in SCD infants with ASSC. We present a unique, oxygen-mediated spleen-on-a-chip platform to study splenic retention and elimination of RBCs and investigate the homeostatic balance between these functions. Our results provide insights into how splenic sequestration and related crises occur in SCD.

The human spleen is a unique organ that plays an important role in our immune and circulatory systems. The spleen is composed primarily of two distinct functional regions, the red pulp and the white pulp, which are intermingled by the marginal/perifollicular zone (1). It contains complex vascular pathways involving direct and indirect connections. Direct connections exist between the fast perifollicular microcirculation and venous sinuses drained in splenic veins (“closed circulation”), whereas indirect connections exist between red pulp arterioles and veins through the reticular meshwork (“open circulation”) and across the wall of sinuses (1). As much as about 80% of the spleen parenchyma is populated with the red pulp, which mainly comprises the vascular sinuses and cords of Billroth (1, 2). Approximately 3 to 10% of blood from cardiac output flows through the spleen, and about 10% of splenic inflow passes through slow open circulation in the red pulp (1, 3–5).

**Fig. 1.**
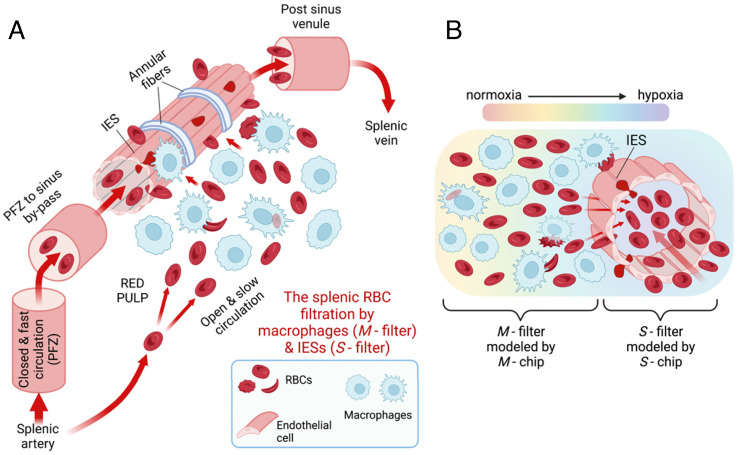
Splenic filtration of altered RBCs. (*A*) Schematic diagram of blood circulation through splenic red pulp, including closed circulation and open circulation. The splenic filtration of altered RBCs is achieved in the open circulation in the red pulp, through macrophages (*M*-filter) and the splenic IESs (*S*-filter). (*B*) Schematic diagram of the oxygen gradient near the sinus. The two structural and functional spleen filters, the *S*-filter and *M*-filter, respectively, are modeled in vitro using the *S*-Chip and *M*-Chip, respectively. Created with BioRender.com.

Splenic filtration of abnormal red blood cells (RBCs) is predominately performed in the open circulation, through macrophage-rich zones (*M*-filter) and across splenic inter-endothelial slits (IESs) in the wall of sinuses (*S*-filter), as shown in Fig. 1*A*. The specialized elongated shape of littoral cells in the splenic sinuses and their three-dimensional (3D) barrel-like structure impose sub-micrometer scale physical barriers or constraints on RBCs navigating the open circulation (6). Prior to returning to the systemic circulation through the IESs across the spleen, circulating RBCs are checked for surface integrity by a scattered collection of resident macrophages (7). These two structural and functional spleen filters, *S*-filter and *M*-filter (Fig. 1*B*), sustain the remarkable capacity of the spleen to retain and destroy abnormal RBCs. Consequently, they contribute to the fine balance between RBC production in the bone marrow and the removal of abnormal RBCs from the blood circulation (1).

Healthy human RBCs (AA RBCs) have an average life span of 100 to 120 d (8), indicating that about 1% of the RBCs are recycled each day by the human body (9). Senescent RBCs typically have reduced deformability (10) and send signals to macrophages by expressing higher phosphatidylserine (PS), higher band-3, and reduced CD47 levels (11, 12), on their external cell surface. Senescent RBCs could exhibit a higher propensity to be sequestered at the IESs due to their reduced deformability and then cleared by macrophages, or trapped by adhesion and phagocytosed in the meshwork (13). Here, a balance between RBC retention rate and RBC elimination rate (by post-retention processing) should be dynamically maintained to ascertain homeostasis. Such homeostatic balance, however, can be severely disrupted due to hemolytic disorders, resulting in serious, and sometimes life-threatening, complications such as splenomegaly and/or hypersplenism (14). As a result, the RBC retention rate can significantly surpass the RBC post-retention elimination rate in the spleen. It is, therefore, important to investigate systematically and quantitatively both the RBC retention rate and the elimination rate in the spleen, and to compare their relative changes, as a function of disease state, with the baseline condition of healthy hemostasis.

The spleen is generally in a hypoxic (low oxygen level) condition owing to its slow and open blood circulation in the red pulp (see the brief review of splenic oxygen level and transit time/velocity of RBCs in *SI Appendix*, Table S2). An oxygen gradient exists in the spleen from locations nearest to the arteriolar end of capillaries to the distal locations in the proximity of the IESs, as shown schematically in Fig. 1*B* (15). Under normal circumstances, hypoxia is mild due to continuous oxygen delivery by the RBCs through splenic circulation. However, it deepens with the reduction in oxygen delivery arising from the obstruction of RBC flow or anemia in many hemolytic blood disorders. For instance, in sickle cell disease (SCD), such obstruction/anemia-induced local hypoxia may in turn trigger sickling of homozygous sickle cell disease (HbSS) RBCs (SS RBCs) and subsequently lead to further reduction in deformability and increase in the expression of adhesion molecules (16, 17). Therefore, sickled RBCs have a higher propensity to be retained by the venous sinuses as well as cords of Billroth, thereby contributing further to a reduction in the oxygen level in the spleen (18). The splenic retention of SS RBCs depends on a trade-off between the local oxygen level (which determines the sickling kinetics) and the transit time of RBCs through the spleen (19). Excessive retention of stiff and sickled SS RBCs in the patient’s spleen has been considered a dominant cause of acute splenic sequestration crisis (ASSC), a life-threatening complication, in SCD (17, 20, 21). This process might involve a vicious cycle: the more RBCs the spleen traps, the larger the spleen grows, and the larger the spleen grows, the deeper the hypoxia is, resulting in more and more sickled SS RBCs that are consequently being trapped and destroyed. Indeed, following splenectomy in young SCD children with still functional spleen, sickled RBCs have been found retained and congested upstream of IESs during ASSC (17, 22, 23). On the other hand, surface modulations such as PS externalization (24), decreased levels of CD47 (25), and elevated binding of autologous immunoglobulin (26), as well as increased membrane rigidity of sickled RBCs may also promote the retention and elimination of SS RBCs by the splenic macrophages (27–29). From these considerations, we postulate that both increased mechanical retention and hyperactive phagocytosis elimination of abnormal RBCs can exacerbate significantly under some extreme conditions such as hypoxia in SCD. These factors could, in turn, play a key role in disrupting the homeostatic balance thereby causing spleen dysfunction in hemolytic disorders.

Recent in vitro studies based on microfluidic spleen-on-a-chip platforms, which simulate the micro-constrictions of IESs and hydrodynamic conditions, have advanced the functional study of RBC filtration in the spleen (30–33). However, to our knowledge, no prior in vitro assays have effectively integrated a controlled gaseous microenvironment within a microfluidic system to enable the quantitative investigation of the hypoxic effect on splenic retention and post-retention elimination of RBCs, especially for SS RBCs. Moreover, there is a compelling need for an in vitro assay that elucidates the mechanisms underlying the interaction of RBCs with splenic phagocytes during the low-velocity microcirculation through the red pulp. Most existing erythrophagocytosis (RBC elimination) assays measure phagocytic activity in a static condition, which does not faithfully replicate in vivo conditions (27, 28). To this end, the development and validation of an oxygen-mediated in vitro assay for investigating the kinetics of both splenic retention of RBCs and erythrophagocytosis under hypoxia are critically needed for a better understanding of the mechanisms responsible for splenic functions in physiology and disease.

Here we present a general microfluidic platform to systematically probe the retention and elimination functions undertaken by IESs and macrophages in the human spleen, by developing and validating two functional modules of an oxygen-mediated spleen-on-a-chip. This platform entails the *S*-Chip and the *M*-Chip, which model the *S*-filter for RBC retention through splenic IESs and the *M*-filter for RBC adhesion and elimination by splenic resident macrophages, respectively. While the microfluidic platform and assays presented in this work can, by design, potentially provide mechanistic insights into a wide spectrum of hereditary and acquired human diseases, we focus particular attention here on the study of homeostatic processes in SCD. We make comparisons with healthy subjects as a negative control group. We additionally use heated AA RBCs as a positive control, while considering it as a generic model for exploring different controlled concentrations of altered RBCs in hemolytic disorders. We demonstrate that our microfluidic platform can also be used to mimic in vitro the two major components of the spleen filtering unit, namely surface sensing by macrophages and mechanical sensing by splenic IESs under controlled oxygen pressure. We further show that this approach enables systematic investigations of the cellular mechanisms underlying anemia and ASSC in SCD, while also providing potential pathways to explore, with appropriate modifications, splenomegaly and hypersplenism in other diseases such as *Plasmodium falciparum* malaria.

## Results

### Mechanical Retention of RBCs by IESs on the *S*-Chip Module.

**Fig. 2.**
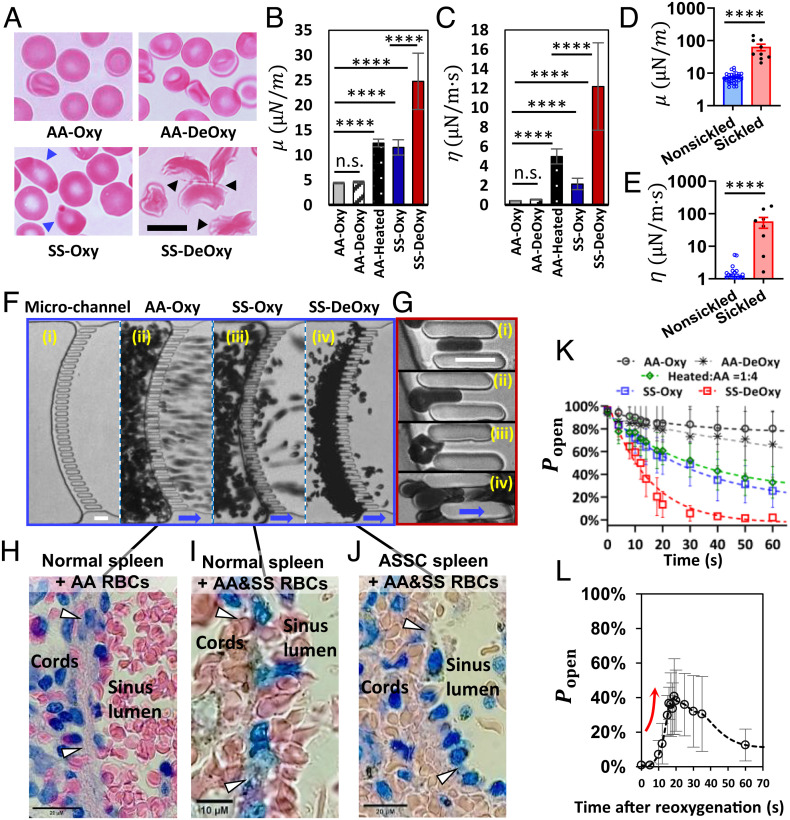
*S*-Chip module—modeling filtration of RBCs through splenic IESs. (*A*) Microscopic images (colorized here for presentation and clarity) of healthy (AA) and sickle (SS) RBCs under oxygenation (Oxy) and deoxygenation (DeOxy) conditions. Blue arrows denote the irreversibly sickled SS RBCs (ISCs) under oxygenation and black arrows denote the sickled SS RBCs under deoxygenation. (Scale bar, 10 µm.) Averaged values of (*B*) shear modulus *µ* and (*C*) shear viscosity *η* measured from individual AA-Oxy RBCs (n = 171), AA-DeOxy RBCs (n = 171), AA-Heated RBCs (n = 102), SS-Oxy RBCs (n = 68), and SS-DeOxy RBCs (n = 52). Error bars indicate the Standard Error of Mean. *P < 0.05, **P < 0.01, ***P < 0.001, ****P < 0.0001, n.s., not significant. Comparisons of (*D*) shear modulus *µ* and (*E*) shear viscosity *η* in non-sickled and sickled RBCs under deoxygenation. Error bars indicate the Standard Deviation (SD). *P < 0.05, **P < 0.01, ***P < 0.001, ****P < 0.0001, n.s., not significant. (*F*) RBC filtration through a microfluidic channel with biomimetic splenic slits. Microfluidic channel (*i*), filtration of AA-Oxy RBCs (*ii*), filtration of SS-Oxy RBCs (*iii*), and filtration of SS-DeOxy RBCs (*iv*). (Scale bars, 10 µm.) Blue arrows denote the flow direction. (*G*) Representative microscopic images of an AA-Oxy RBC traversing the slit (*i*), a SS-Oxy RBC largely elongated and blocked after entering the slit (*ii*), a sickled SS RBC blocked by the slit (*iii*), and multiple SS RBCs clustered in a slit under oxygenation (*iv*), respectively. (Scale bars, 10 µm.) Blue arrows denote the flow direction. (*H*–*J*) Histological images of red pulp in human spleens: (*H*) normal human spleen perfused ex vivo with AA RBCs without congestion in the red pulp, (*I*) normal spleen perfused with a mixture of AA and SS RBCs with significant RBC congestion and focal sickling, and (*J*) ASSC spleen after splenectomy containing AA and SS RBCs (the patient was transfused before splenectomy), showing severe RBC congestion and significant sickling. White arrows denote the blood flow direction from splenic cords to sinuses. See *SI Appendix, Ex Vivo Experiments on Human Spleens* for details. (*K*) Experimental data of mechanical retention for different RBC samples/subpopulations: AA-Oxy RBCs (n = 7), AA-DeOxy RBCs (n = 5), a mixture of AA-Heated and AA RBCs in a ratio of 1:4 (n = 8), SS-Oxy RBCs (n = 11), and SS-DeOxy RBCs (n = 8). Time t = 0 refers to the point just prior to the first retention of RBCs by the IESs. Each data point represents averaged values of the instantaneous proportion of slits that remained open ($P_{open}$) in the microfluidic channel at different time points. Error bars indicate SD. Dashed curves represent exponential fit to experimental data. (*L*) Alleviated retention of SS-DeOxy RBCs upon reoxygenation after blockage in the micro-slits (n = 5). The red arrow denotes a rapid increase in the proportion of slits that remained open after reoxygenation.

The mechanical retention of RBCs by the IESs in the spleen is influenced by the shape and mechanical properties of individual RBCs (34–37). Sickled or elongated RBCs have been found retained by IESs in SCD patients with ASSC (17, 22). In order to differentiate morphologies of SS RBCs with those of AA RBCs, we studied both AA and SS RBCs under oxygenated (Oxy) and deoxygenated (DeOxy) conditions using a sickling kinetics assay developed in our earlier work (38, 39). The heights of the cell channel and gas channel of the *S*-Chip in the present study are the same as in these prior designs (38, 39). The AA RBCs showed no obvious changes in their typical biconcave disc shapes before and after 2 min of deoxygenation (decreasing from 20% O_2_ to 2% O_2_). By contrast, most of the SS RBCs formed crescent shapes with spiky spicules and dark coarse texture after deoxygenation, which are visually identified as sickled SS RBCs. When SS RBCs were reoxygenated, most of the sickled SS RBCs could resume (unsickle) to their regular shapes due to depolymerization of sickle hemoglobin (HbS) while the so-called irreversibly sickled cells (ISCs) keep their typical sickled shape (Fig. 2*A*). Note that HbS in ISCs can still polymerize and depolymerize upon deoxygenation and reoxygenation, respectively. Here in this study, we denote all SS RBCs (including ISCs) under normoxia as “non-sickled” and categorize those ISCs with no obvious intracellular HbS polymerization under hypoxia as part of the non-sickled subpopulation.

To compare the mechanical properties of AA and SS RBCs under Oxy and DeOxy conditions, we characterized the shear modulus (*μ*) and shear viscosity (*η*) values of the membranes of individual RBCs in each condition using the electrodeformation method under controlled oxygen pressure, as described in our previous work (40–42). We also measured heated AA RBCs as positive control and to present sample conditions with different controlled levels of altered RBCs because they are known to have decreased deformability and show higher mechanical retention rates through narrow slits (34, 43) (**SI Appendix*, Sample Preparation for In Vitro Experiments*). Fig. 2 *B* and *C* show comparisons of averaged *μ* and *η* values measured from AA-Oxy RBCs (n = 171), AA-DeOxy RBCs (n = 171), AA-Heated RBCs (n = 102), SS-Oxy RBCs (n = 68), and SS-DeOxy RBCs (n = 52). The *μ* and *η* values for AA RBCs didn’t show statistically significant changes after 2 min of deoxygenation compared to the control values in the Oxy state (4.5% increase in *μ* and 31.3% increase in *η*, *P* > 0.05). The *μ* and *η* values measured in the AA-Heated RBCs were significantly higher than those in AA-Oxy RBCs (~threefold in *μ* and ~10-fold in *η*, with *P* < 0.0001), which is consistent with expected trends (44). Similarly, SS-Oxy RBCs showed significant increases in *μ* and *η* values when compared with the control values of AA-Oxy RBCs (~threefold in *μ* and ~fivefold in *η*, with *P* < 0.0001). By contrast, the increase in the averaged *μ* and *η* values was more prominent for SS-DeOxy RBCs relative to AA-Oxy RBCs (~sixfold in *μ* and ~30-fold in *η*, with *P* < 0.0001). Additionally, SS-DeOxy RBCs showed marked heterogeneity in *μ* and *η* values, which might be attributed to the large differences among the SS RBCs with wide variations in their propensity for sickling under deoxygenation (45). To confirm this, we compared the *μ* and *η* values of the non-sickled subpopulation with those of the sickled subpopulation from DeOxy SS RBCs (Fig. 2 *D* and *E*). We found that both *μ* and *η* values of the sickled RBCs were significantly higher than those for non-sickled RBCs (10~100-fold, with *P* < 0.0001). This suggests that the retention rate of SS RBCs is greatly dependent on the sickled fraction under hypoxia.

We further investigated the mechanical retention of RBCs from five different groups, comprising AA-Oxy RBCs, AA-DeOxy RBCs, a mixture of AA-Heated RBCs and AA RBCs in a ratio of 1:4 (Heated:AA = 1:4 in numbers), SS-Oxy RBCs, and SS-DeOxy RBCs. Here, we use the mixture of AA-Heated RBCs and AA RBCs (1:4) to mimic a generic hemolytic disorder containing a significant percentage of altered RBCs (~20%). We performed the perfusion of RBCs from each group through a microfluidic channel incorporating a curved row of 32 narrow micro-gates (3 µm wide, 5 µm deep, and 15 µm long) simulating the overall geometry of IESs in the human spleen (Fig. 2 *F*, *i*). The transient deoxygenation of cells was controlled by exchanging the oxygen level from the upper layer of gas channel in a double-layer microfluidic device (38). To achieve a moderate retention rate of RBCs and to normalize the starting condition across different blood samples, all RBC samples were diluted to a 10% hematocrit with phosphate-buffered saline (PBS) containing 1% (w/v) bovine serum albumin. Perfusion was achieved by connecting the microfluidic channel to an external hydraulic column to generate gravity-driven flow of RBC suspension through the slits under an equivalent pressure difference of ~3 kPa. The right three panels in the blue frame of Fig. 2*F* show a comparison of filtration behavior among AA-Oxy RBCs, SS-Oxy RBCs, and SS-DeOxy RBCs. During transient perfusion, we observed that most AA RBCs rapidly traverse the micro-slits, and only a small fraction of the slits in the channel were obstructed (Fig. 2 *F*, *ii* and Movie S1). For SS-Oxy RBCs, a much larger fraction of the slits was eventually blocked (Fig. 2 *F*, *iii* and Movie S2). By contrast, most of SS RBCs were sickled and consequently stiffened while flowing upstream of the micro-slits during deoxygenation and were quickly retained at the slits. Only a few cells that showed delayed sickling downstream could pass through the slits. The upstream cell flow slowed down significantly due to the quick blockage of the slits (Fig. 2 *F*, *iv* and Movie S3).

As shown in Fig. 2*G*, we observed the rheological characteristics of individual RBCs in the individual slits under different conditions. Fig. 2 *G*, *i*–*iv* show an AA-Oxy RBC traversing through a slit; a SS-Oxy RBC, a sickled SS-DeOxy RBC, and a cluster of multiple SS-Oxy RBCs trapped by a single slit, respectively. As shown in Fig. 2 *H*–*J*, similar contradistinction of splenic filtration behaviors under different conditions was also observed from the histological analysis of human spleens. Fig. 2 *H* and *I* show the ex vivo healthy human spleen perfused with AA RBCs, and a mixture of AA and SS RBCs, respectively. Similar to our observations in the in vitro experiments, there is no RBC congestion as shown in Fig. 2*H*. Fig. 2*I* reveals significant RBC congestion and isolated sickling (not widespread sickling) in the red pulp. Fig. 2*J* shows pronounced sickling and severe RBC congestion upstream of the IESs in the spleen of an ASSC child’s spleen underwent a partial septectomy (17, 46, 47) (see *SI Appendix*, *Ex Vivo Experiments on Human Spleens* for details).

Fig. 2*K* shows experimental measurements of the averaged fraction (in %) of slits that remained open without being blocked by the RBCs as a function of time for different conditions: AA-Oxy RBCs (n = 7), AA-DeOxy RBCs (n = 5), Heated:AA = 1:4 RBCs (n = 8), SS-Oxy RBCs (n = 11), and SS-DeOxy RBCs (n = 8). Here the hypoxia (DeOxy) condition has an oxygen concentration of 2% O_2_. The retention rates for the RBC groups during the perfusion process increase in the following order: AA-Oxy RBCs < AA-DeOxy RBCs < Heated:AA = 1:4 RBCs ≈ SS-Oxy RBCs < SS-DeOxy RBCs. The difference between AA-Oxy RBCs and AA-DeOxy RBCs is much smaller than that between SS-Oxy RBCs and SS-DeOxy, suggesting that the effect of hypoxia on mechanical retention of SS RBCs is more pronounced than that for AA RBCs. The increase in the retention rate of SS RBCs when deoxygenated can be attributed to the pronounced stiffening of sickled RBCs due to the intracellular polymerization of HbS. The sickling process of SS RBCs is reversible to some extent following reoxygenation. Thus, we investigated the retention behavior of SS-DeOxy RBCs upon reoxygenation after all the micro-slits in the microfluidic channel were blocked by cells. Interestingly, we observed that many of the blocked micro-slits reopened upon reoxygenation, and then gradually became obstructed again by cells from upstream (Movie S4). The percentage of open slits increased from 0% to about 40% within 20 s, and then gradually decreased to below 20% about 50 s thereafter (Fig. 2*L*). This result suggests that an instant increase in oxygen level could substantially reverse the mechanical retention of SS-DeOxy RBCs at the splenic slits and unclog them. Additionally, the retention behavior under mild hypoxia (5% O_2_) was found to be similar to that under normoxia (*SI Appendix*, Fig. S3 and *Splenic Slit Retention Behavior Under Mild and Severe Hypoxia*).

To squeeze through the micro-slits, individual RBCs must deform and stretch to large strains due to physical constrictions at the slits. We assume that the passable cells must be able to fully enter a relatively long slit and quasi-statically reach a maximum extension (*L*_S_) before exiting the slit (*SI Appendix,* Fig. S1). Under a given pressure differential, the critical condition for the cells traversing the micro-slits is dependent on the shear modulus of individual RBCs. We estimated a threshold shear modulus (*µ*_c_) of ~14.6 μN/m for individual RBCs from our applied pressure difference based on the Young–Laplace equation (48). This value agrees well with the value obtained using dissipative particle dynamics (DPD) simulations. Moreover, the equivalent critical conditions for different slit geometries can also be estimated through DPD simulations. For instance, the slit geometry used in the present study (3 µm wide, 5 µm deep, and 15 µm long) is found to have a comparable critical condition for RBC retention as the slit geometry that represents an IES. (More details on estimating the critical shear modulus *µ*_c_ can be found in *SI Appendix*, *Modeling of Mechanical Filtration of RBCs by Micro-Slits/IESs*.) With this simple analysis focusing on critical membrane stiffness or a more rigorous analysis for estimating the critical passage conditions, RBCs can be classified as “slit-passable” (the subpopulation that is capable of flowing through the slits) and “slit-unpassable” (the subpopulation that will be retained by the slits). The fraction (F) of slit-unpassable or “slit-retained” RBCs within the total population of RBCs can then be determined. Assuming F is dominated by a threshold shear modulus (*µ*_c_), we have:

[1] $$F=\frac{N_{\mu >\mu_{c}}}{N_{\mathrm{total}}}.$$

Values of F estimated from the results shown in Fig. 2*B* are: 0.58% for both AA-Oxy RBCs and AA-DeOxy RBCs, 6.1% for Heated: AA=1:4 RBCs, 22.1% for SS-Oxy RBCs, and 64.3% for SS-DeOxy RBCs. Note that the critical conditions for RBC blockage/passage may vary with the structural constraints of IESs in the human splenic red pulp (34). We can account for such variations by adjusting the design of the slits in the microfluidic channel or by changing the pressure difference across the slits in the experiments.

*SI Appendix,* Fig. S1*F* provides our simulation results of the estimated proportion of slits that remained open (Popen) as a function of time (t) for different RBC subpopulations based on a simple analytical RBC-filtration model. (see *SI Appendix*, *Modeling of Mechanical Filtration of RBCs by Micro-Slits/IESs* for details). We find that the retention rate of RBCs increases with the fraction of slit-unpassable RBCs. This estimation provides a quantitative correlation between the probability of mechanical retention and stiffness values of individual RBCs. The trends for the change in retention behavior of RBCs measured from our experiments agreed generally with the simulation results for each cell type. Discrepancies between the estimated trends and experimental results of the retention rate in SS RBCs could arise from the large heterogeneity in shape and properties within the cell population, as well as the variations in the sickled fractions under deoxygenation among different SS samples (38, 49). While more sophisticated models that account for RBC shapes and heterogeneities can be used to improve the agreement with experiments, the overall general agreement of our simple analytical model indicates that cell stiffness is a major factor in determining micro-slit blockage/passage.

### Study of Adhesion of RBCs Using the *M*-Chip Module.

**Fig. 3.**
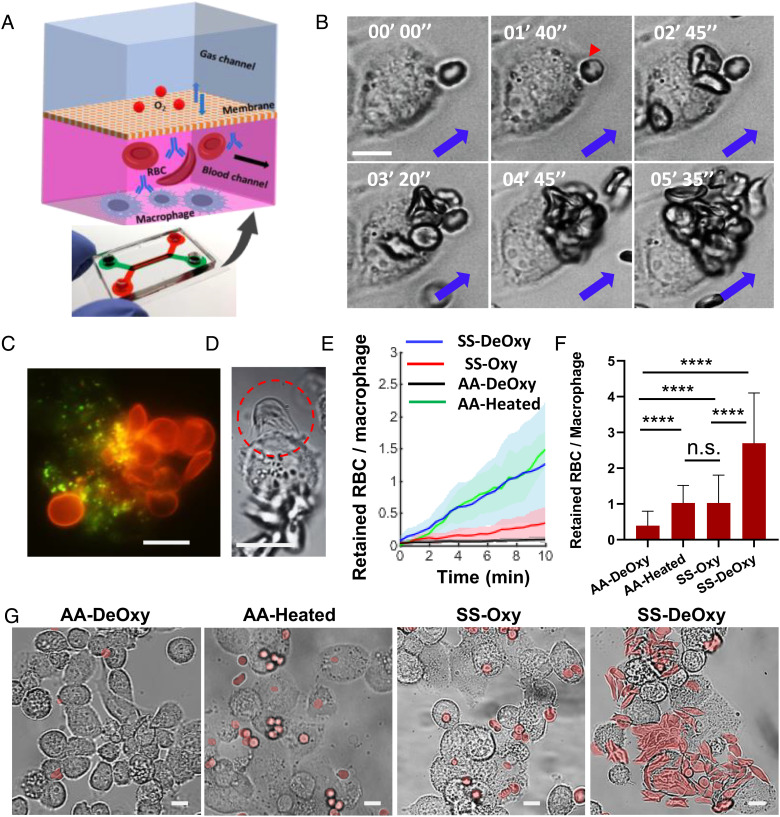
*M*-Chip module—modeling retention of SS RBCs by resident macrophages in the spleen under the flow condition. (*A*) The microfluidic chip used for the M-filter study. Schematic shows the functionalized microchannel for blood perfusion over THP-1 macrophages (MФ) cultured on the bottom wall, equipped with a top layer of gas channel for oxygen exchange. (*B*) Time-lapse images showing a macrophage-retention process of SS RBCs being progressively adhered on a representative individual MФ under hypoxia. The red arrow denotes an RBC which is in the initial stage of being engulfed by the macrophage after adhesion. Blue arrows denote the flow direction. (*C*) A representative fluorescence microscopic image showing multiple PKH26-stained SS RBCs (red) retained on the surface of a PKH67-stained MФ (green). (*D*) A sickled RBC strongly attached to an MФ with its sharp spicules of polymerized HbS protrusion (circled by the red dashed line). (*E*) The average number of retained RBCs per macrophage as a function of time with the progression of RBC retention for the AA-DeOxy (n = 5), AA-Heated (n = 5), SS-DeOxy (n = 9), and SS-DeOxy RBCs (n = 8). Timepoint t = 0 is defined by the time right before the first retention of any RBCs by the macrophages. (*F*) Comparison of the average number of retained RBCs per macrophage after 30 min of perfusion including a high flow rate (10×) flush step, among AA-DeOxy RBCs (n = 5), AA-Heated RBCs (n = 5), SS-DeOxy RBCs (n = 9), and SS-DeOxy RBCs (n = 8). (*G*) Microscopic images of RBCs retention by MФ after 30 min of perfusion for AA-DeOxy RBCs, AA-Heated RBCs, SS-Oxy RBCs, and SS-DeOxy RBCs. RBCs are denoted in light red color. (Scale bars, 10 µm.) Error bars indicate the SD. *P < 0.05, **P < 0.01, ***P < 0.001, ****P < 0.0001, n.s., not significant.

In addition to the mechanical retention by the IESs, adhesion of SS RBCs to the reticular meshwork or macrophages could be another critical factor involved in splenic vaso-occlusion (1). The initial adherence of RBCs to the surface of macrophages must play an important role in subsequent erythrophagocytosis. We investigated the adhesion behavior of SS RBCs on THP-1 macrophages (a human monocytic cell line used extensively to study monocyte/macrophage behaviors and functions) under shear flow conditions (~0.003 Pa pressure with a flow rate of ~0.1 mL/h) using the *M*-Chip module (Fig. 3*A*). The *M*-Chip consisted of two layers of microfluidic channel comprising a blood channel and a gas channel. The oxygen level over the RBCs and macrophages in the blood channel was mediated by exchanging oxygen within the gas channel through a gas-permeable polydimethylsiloxane (PDMS) membrane. We observed a marked increase of adhesion in SS-DeOxy RBCs during continuous blood perfusion on the macrophages compared to that in SS-Oxy RBCs.

Fig. 3*B* shows time-lapse images of a macrophage-retention process of SS RBCs being progressively adhered on the surface of an individual macrophage under hypoxia (Movie S5). We observed that the adhesion-retention process of SS RBCs commenced with the initial attachment of a single RBC on the macrophage. The adhered cells subsequently started to be engulfed by the macrophage after a minute (see for example the cell denoted by the red arrow in Fig. 3*B*). RBCs from upstream tended to be congested in the vicinity of sites where prior RBCs adhered to the surfaces of macrophages. This process is seemingly aided by several factors: 1) The initially adhered RBCs could further stimulate specific surface receptors of macrophages, thereby upregulating their phagocytic response to subsequent RBCs; 2) The RBCs from upstream cluster on the surface of previously attached RBCs due to agglutination; 3) The adhered RBCs formed physical barriers narrowing the pathway for subsequent RBCs from upstream to pass through. To preclude severe clogging of the channel, we used diluted blood samples and focused on the short-term differences among different groups. By labeling SS RBCs and macrophages using PKH26 dye (red) and PKH67 dye (green), we confirmed that most adhered RBCs were eventually internalized by the macrophages during the continuous adhesion process, and there were also a few instances of RBC-to-RBC adherence (Fig. 3*C*). Due to the limitation of two-dimensional (2D) imaging, we were not able to clearly observe how RBCs interacted with the surface of the macrophage especially after a number of RBCs clumped together. However, we could still see the attachment of RBCs on the edge of macrophages. For instance, Fig. 3*D* shows a sickled SS RBC under hypoxia strongly attached to a macrophage with its sharp spicules of polymerized HbS protrusion. This SS RBC maintained its firm adhesion even though we increased the shear flow rate up to ~1 mL/h (Movie S6).

To quantify the dynamic process of RBC retention due to adhesion on the macrophages, we counted the number of attached RBCs on all the macrophages in the field of view during continuous blood perfusion over 10 min. Fig. 3*E* shows the comparison of average number of attached RBCs per macrophage as a function of time from AA-DeOxy RBCs, AA-Heated RBCs, SS-Oxy RBCs, and SS-Oxy RBCs. In contrast to the control (AA-DeOxy RBCs), SS-Oxy RBCs showed higher retention rate under normoxia, and SS-DeOxy RBCs exhibited the highest adhesion rate under hypoxia, which is similar to that of the AA-Heated RBCs. We then stopped blood perfusion and switched to the flow of PBS solution with an order-of-magnitude greater flow rate of 1 mL/h for an additional 20 min. The residual RBCs upstream in the channel continued to be perfused. Consequently, more RBCs were attached to the macrophages while most unattached RBCs were washed out by the higher shear flow. SS-DeOxy RBCs showed the most significant increase in the average number of attached RBCs per macrophage within 30 min compared to the other groups. The number of RBCs attached to the macrophage increased in the following order: AA-DeOxy RBCs (n = 5) < AA-Heated RBCs (n = 5) ≈ SS-Oxy RBCs (n = 9) < SS-DeOxy RBCs (n = 8) (Fig. 3*F*). Comparing the results shown in Fig. 3*E* with those in Fig. 3*F*, AA-Heated RBCs appear to have weaker adhesion to macrophages than SS-DeOxy RBCs. Here, an order of magnitude faster flow appears to have dislodged and detached some previously adhered heated RBCs. Fig. 3*G* shows the representative images of the adherence of RBCs on macrophages after the conclusion of the entire process for AA-DeOxy RBCs, AA-Heated RBCs, SS-Oxy RBCs, and SS-Oxy RBCs. The results suggest that the hypoxic condition can significantly increase the adhesion of SS RBCs to macrophages, thereby exacerbating congestion of SS-RBCs in splenic open circulation, in addition to IES filtration.

### Study of Phagocytosis of RBCs Using the *M*-Chip Module.

**Fig. 4.**
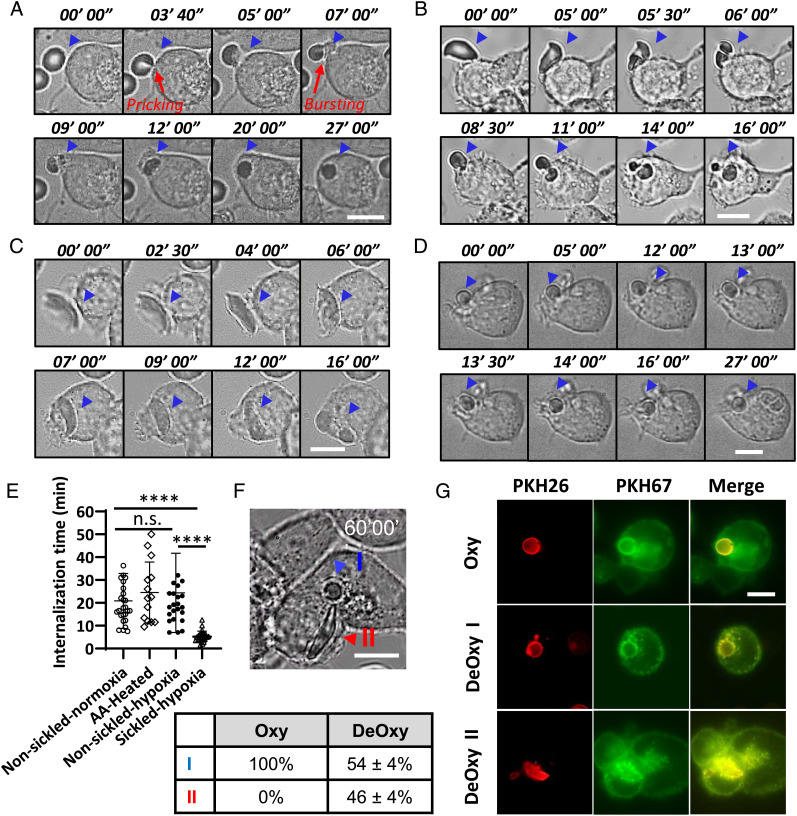
Sickled SS RBCs show faster phagocytic uptake but slower digestion. Phagocytic activity of (*A*) a non-sickled SS RBC under normoxia, (*B*) an irreversibly sickled SS RBC under normoxia, (*C*) a sickled SS RBC under hypoxia, and (*D*) a heated AA RBC, by human-derived THP-1 macrophages (MФ). Time-lapse imaging begins with initial attachment between RBC and MФ and ends on complete engulfment. (*E*) Comparison of internalization time between (non-sickled) SS RBCs under normoxia, AA-heated RBCs, non-sickled SS RBCs under hypoxia, and sickled SS RBCs under hypoxia. (*F*) A representative microscopic image showing distinct phagosomes of vesicles (denoted by the blue arrow, type I) and a sickled SS RBC (denoted by the red arrow, type II) in an identical MФ under hypoxia. The table underneath indicates the fractions of these two types of cells (type I and type II) after 60 min of internalization in MФ. (*G*) Fluorescence microscopic images of a phagocytized SS RBC under normoxia (*Upper*), fused SS RBC under hypoxia, and unfused sickled SS RBC under hypoxia (*Lower*). RBCs and MФ were labeled by PKH26 (red) and PKH67 (green), respectively. (Scale bars, 10 µm.) Error bars indicate the SD. *P < 0.05, **P < 0.01, ***P < 0.001, ****P < 0.0001, n.s., not significant.

Next, we investigated the engulfment process of SS RBCs by the THP-1 macrophages under both oxygenated and deoxygenated conditions using the *M*-Chip module. The SS-Oxy RBCs with typical biconcave shape showed no distinct difference from those of AA RBCs described in previous work (28). As shown in Fig. 4*A* and Movie S7, the phagocytosis of a SS RBC under normoxia revealed several typical steps: i) The macrophage and the target RBC were attached to each other after a period of initial interaction. ii) The macrophage pinched the RBC membrane and formed a semi-conical synapse, and then the RBC was pricked and burst into a spherocyte with a significantly smaller diameter. iii) The macrophage pseudopods extended and surrounded the RBC to form a phagocytic cup. iv) The RBC was fully engulfed by the macrophage. v) The RBC was further digested and formed the phagosome. Such phenomena were repeatedly observed in our experiments, regardless of whether the SS RBC had a regular biconcave disc shape or an abnormal ISC shape (Fig. 4*B* and Movie S8). We have measured the internalization time from the initial attachment of RBCs to the surface of the macrophage in step (i) to the full engulfment by the macrophage after step (iv) during the phagocytosis process of RBCs. In some cases, this step-by-step progress appeared to prolong the total engulfment time of SS-Oxy RBCs. We observed that some of the SS-Oxy RBCs were not fully internalized even after 50 min (*SI Appendix,* Fig. S2 and Movie S9). Under hypoxia, the non-sickled SS-DeOxy RBCs (with no visible polymerization of HbS) exhibited an engulfment process similar to that of SS-Oxy RBCs (Movie S10). By contrast, those sickled SS-DeOxy RBCs (with HbS polymerization) showed a much faster engulfment rate compared to the SS-Oxy RBCs and non-sickled SS-DeOxy RBCs (Fig. 4*C* and Movie S11). We found that most of these sickled SS RBCs skipped the step of pricking and bursting [step (ii)] and appeared to be “wolfed down” by the macrophage during the engulfment process. Apparently, the polymerized HbS fibers inside the sickled SS RBCs make them more rigid and harder to be pricked by the macrophages than those non-sickled RBCs. This behavior is of particular interest and is a likely reason for the accelerated engulfment of sickled SS-DeOxy RBCs compared with the SS-Oxy RBCs and non-sickled SS-DeOxy RBCs. However, even though most of the AA-heated RBCs already transformed into spherocytes and could skip the pricking and bursting step [step (ii)] found in the case of non-sickled RBCs, the overall internalization time did not shorten compared to those non-sickled RBCs (Fig. 4*D* and Movie S12).

We measured the internalization time for these three types of SS RBCs and the AA-heated RBCs (Fig. 4*E*). The results show that the SS-Oxy RBCs, AA-heated RBCs, and non-sickled SS-DeOxy RBCs show similar averaged internalization time of 20.9 ± 12.1 min (n = 26), 23.7 ± 8.3 min (n = 5), and 24.4 ± 17.3 min (n = 25), respectively, with no statistically significant difference. By contrast, the averaged internalization time of the subpopulation of sickled SS-DeOxy RBCs is significantly shortened at 5.2 ± 2.4 min (n = 26). Additionally, we found that most of these sickled SS-DeOxy RBCs took a prolonged period to be digested. Interestingly, in contrast to the fact that all the phagocytosed SS-Oxy RBCs formed the phagosome and fused as vesicles before or after the internalization process (type I, as denoted by the blue arrow in Fig. 4*F* and Movie S11), a significant portion of SS-DeOxy RBCs kept their polymerized hemoglobin and sickled shape in the interior of the macrophages for up to ~3 h (type II, as denoted by the red arrow in Fig. 4*F* and Movie S13). This is likely due to the polymerized HbS fibers inside the RBCs, making these sickled SS RBCs much more rigid and harder to be fragmented and digested by the macrophages. We thus evaluated the proportion of these two types of scenarios after 1 h of incubation under the oxygenated and deoxygenated conditions, respectively. We found those type II cells account for 46 ± 4% of the internalized SS-DeOxy RBCs (n = 9) *vs*. 0% of the internalized SS-Oxy RBCs (n = 4) in a series of experiments. To confirm those type I and type II cells were indeed internalized within the macrophages, we also labeled SS RBCs and macrophages using PKH26 dye (red) and PKH67 dye (green) under the oxygenated and deoxygenated conditions, respectively (Fig. 4*G*).

**Fig. 5.**
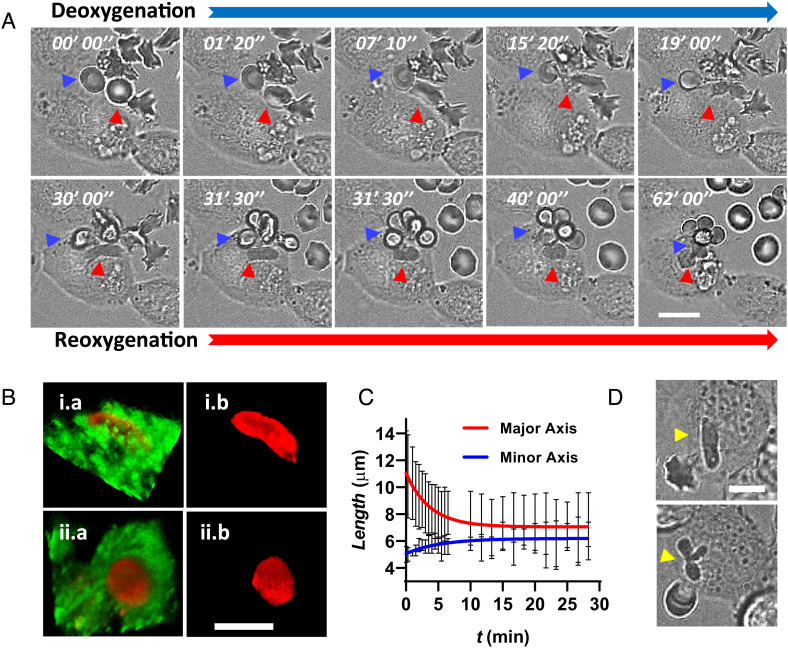
Reoxygenation triggers intra-macrophagic fragmentation of engulfed sickled SS RBCs. (*A*) Time-lapse imaging of phagocytic activity of SS RBCs during deoxygenation (blue notched arrow) and reoxygenation (red notched arrow). The reoxygenation process starts at t = 30″00′. The blue arrow and red arrow denote a typical non-sickled SS RBC and sickled SS RBC, respectively. (*B*) Representative 3D confocal images of sickled SS RBCs (red) before (i) reoxygenation and (ii) after 10 min of reoxygenation in the macrophage (green). *Left* panels show the merged images (*a*), and *Right* panels show the separated red channel (*b*). (*C*) Lengths of major axis and minor axis in 2D images of internalized RBCs as a function of time during the reoxygenation process. Error bars indicate SD of the mean (n = 5). The solid lines represent the best-fit exponential curves. (*D*) Representative microscopic images of a fragmented sickled RBC after 20 min of reoxygenation process. The yellow arrows denote the RBC before reoxygenation (*Upper*) and after reoxygenation (*Lower*). (Scale bars, 10 µm.) Error bars indicate SD.

In a separate set of experiments, we switched the gas condition back to oxygenation after incubating SS RBCs and macrophages together in the deoxygenated condition for 30 min. We observed the typical phagocytosis processes for two different types of cells: a non-sickled SS RBC (as denoted by the blue arrow) and a sickled SS RBC (as denoted by the red arrow) during the deoxygenation and reoxygenation processes (Fig. 5*A* and Movie S14). During the deoxygenation process, the sickled SS RBC showed faster internalization compared to the non-sickled SS RBC which was phagocytosed by the same macrophage (Fig. 5*A*). This suggests that the difference in the phagocytosis process between non-sickled SS RBCs and sickled SS RBCs mainly arises from the abnormality of RBCs instead of from the macrophages. Upon reoxygenation, the internalized but unfused SS-DeOxy RBC (type II) quickly changed to a spherical shape within a few minutes and thereafter showed expedited fusion compared to the SS-DeOxy RBCs under deoxygenation. These trends were repeatedly observed in our experiments. Internalized SS RBCs exhibited a volume reduction of more than 30% after 10 min of reoxygenation (n = 3) as found in our 3D volume measurements from the confocal images (Fig. 5*B*). Fig. 5*C* shows the instantaneous variations in the major and minor axes of the internalized RBCs as a function of time after reoxygenation (n = 5). These results indicate that most internalized sickled SS RBCs quickly change to smaller spherocytes within 10 min after reoxygenation. In addition, we also observed that some internalized sickled SS RBCs showed obvious fragmentation upon reoxygenation (Fig. 5*D* and Movie S15).

## Discussion and Concluding Remarks

In the present study, we established an oxygen-mediated spleen-on-a-chip in vitro platform and investigated the roles played by the IESs and macrophages in maintaining the homeostatic balance between retention and elimination of altered RBCs in the human spleen, with a focus on SCD. The key findings are:

1) Compared to AA RBCs, the retention of SS RBCs in micro-slits increases significantly under normoxia (~38-fold), and even more intensely under hypoxia (~111-fold).
2) A simple analytical RBC-filtration model correlating the stiffness of RBCs to their mechanical retention by micro-slits replicates the major experimental trends and provides quantitative insights into the retention of SS RBCs and heated RBCs. This model can be used to estimate splenic retention of altered RBCs due to increased stiffness in other diseases, such as RBCs infected by the major malaria parasite *P. falciparum* (*SI Appendix*, *Mechanical Filtration of P. falciparum-Invaded RBCs by Micro-Slits* for details).
3) Reoxygenation reverses retention of deoxygenated SS RBCs at micro-slits, with almost half of blocked micro-slits reopening within seconds. Unsickling of sickled SS RBCs blocked in the slits is the major mechanism of slits reopening.
4) Compared to AA RBCs, SS RBCs adhere more rapidly and more intensely to macrophages, especially under hypoxia.
5) Those firmly adherent RBCs are phagocytosed subsequently. Under hypoxia, phagocytosis of non-sickled SS RBC is similar to that of AA RBCs and to that of SS RBCs under normoxia. Phagocytosis of SS RBCs sickled under hypoxia differs from that of SS RBCs under normoxia. The engulfment is much faster while the fragmentation/digestion process is much slower with the majority of the sickled SS RBCs retaining their sickled shape for 1 to 3 h or longer after being engulfed.
6) Upon reoxygenation after the engulfment of sickled SS RBCs, phagocytosed sickled SS RBCs unsickle inside the macrophages and then undergo a rapid fusion/fragmentation process.
7) Heated AA RBCs can be used as a generic model for hemolytic disorders in studying splenic RBC retention and elimination. AA RBCs containing 20% heated RBCs show a similar, albeit slightly lower mechanical retention by micro-slits than that of SS RBCs under normoxia. Heated AA RBCs also show rapid adhesion to macrophages under the slow-flow condition, significantly higher than AA RBCs. Additionally, heated RBCs showed similar internalization time compared to the SS RBCs under normoxia.

### Pathogenic Imbalance between Retention and Post-Retention Phagocytic Elimination of Altered RBCs in the Spleen.

**Fig. 6.**
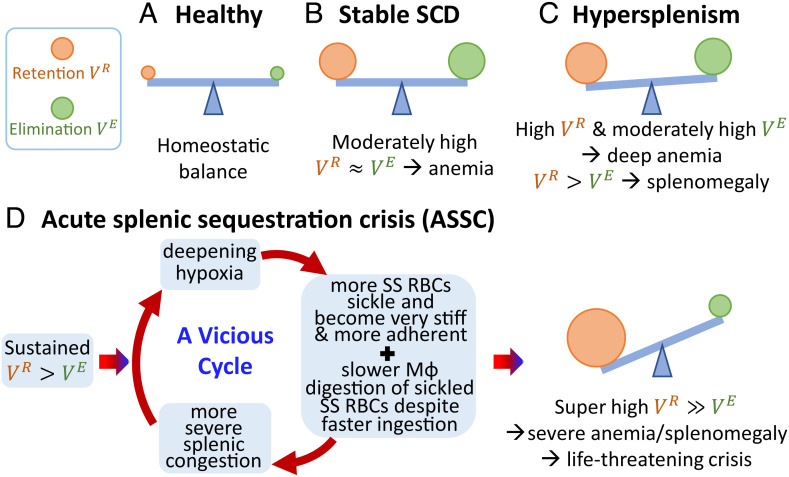
Importance of maintaining the homeostatic balance between splenic retention and phagocytic elimination of altered RBCs. (*A*) Homeostatic balance is maintained in healthy subjects in whom only a small percentage of altered RBCs needs to be eliminated and RBC retention rate and elimination rate are similar ($V^{R}\approx V^{E}$). (*B*) For sickle cell patients in a steady state condition, where anemia is stable and moderate, retention is moderately high, but elimination is similarly increased preventing the occurrence of gross splenomegaly (moderately high $V^{R}\approx$ moderately high $V^{E}$). (*C*) In some patients, elimination is not increased enough to compensate for the moderate increase in retention (high $V^{R}>$ moderately high $V^{E})$ leading to subacute/chronic moderate splenomegaly and deep anemia. (*D*) For sickle cell patients in ASSC, we hypothesize that when the RBC elimination rate cannot keep up with the high retention rate (sustained $V^{R}>V^{E}$). It may induce worsening and eventually severe hypoxia in the spleen, and consequently result in super-fast retention of sickled SS RBCs that will quickly overwhelm the full capacity of splenic elimination by macrophages (Mϕ), with acute and marked splenomegaly and abrupt severe anemia.

At homeostasis**,** the rate of RBC retention ($V^{R}$) and the rate of post-retention elimination ($V^{E}$) need to maintain balance in the spleen, as schematically illustrated in Fig. 6. The RBC retention rate $V^{R}$ includes contributions from the adhesion in the “*M*-filter” and filtering by IESs in the “*S*-filter,” and the speed of elimination is mainly achieved by phagocytosis in the *M*-filter (Figs. 1 and 6). In healthy subjects, the small percentage of senescent RBCs retained by the spleen must be efficiently eliminated by the splenic macrophages, so as to keep its continued filtration function of RBCs in blood circulation (Fig. 6*A*). A recent study reported that many children with SCD have splenomegaly but few have total auto-splenectomy (50). For pediatric SCD patients with some residual spleen function in stable condition, it is likely that the level of hypoxia in the red pulp is not as severe as that in ASSC (see Fig. 6 *B* and *C* for two commonly observed situations with and without splenomegaly). Our *S*-Chip results for SS-Oxy RBCs show that, compared with AA RBCs, there is a much higher percentage of stiff RBCs that cannot pass through IESs (similarly mild retention behavior is observed for SS-DeOxy RBCs under 5% mild hypoxia, see *SI Appendix*, Fig. S3); and our *M*-Chip results suggest that there is a much higher rate of RBC adhesion to macrophages for SS-Oxy RBCs than that for AA RBCs. These results indicate that approximately an order of magnitude higher percentage of altered RBCs is subject to splenic retention (i.e., high $V^{R}$) and elimination (i.e., high $V^{E}$) for SS RBCs under normoxia and mild hypoxia than that for healthy AA RBCs.

The commonly observed anemia in SCD patients may be attributed to the order of magnitude higher splenic retention and elimination rate for SS-Oxy RBCs than that for healthy AA RBCs. When RBC retention rate is approximately the same as the elimination rate $V^{R}\approx V^{E}$, splenomegaly is unlikely to develop. When RBC retention rate becomes faster than elimination rate $V^{R}≳V^{E},$ some degree of splenomegaly or gradually worsening splenomegaly could occur, as observed in 28% in SCD children (50). No severe hypoxia or widespread sickling in the spleen would be expected in most cases. However, about 5 ~ 10% SCD infants develop a life-threatening crisis—ASSC, where significant sickling of SS RBCs can be found (17, 22, 23). Fig. 6*D* schematically illustrates how ASSC could be induced by gradually worsening hypoxia. When RBC elimination rate cannot keep up with the high retention rate (sustained $V^{E}≲V^{R}$), it may trigger a vicious cycle between worsening hypoxia and more severe congestion in the spleen, and consequently result in severe hypoxia and super-fast retention of sickled SS RBCs that will surpass the full capacity of splenic elimination by macrophages.

The *S*-Chip results for SS-DeOxy RBCs show that, compared with AA RBCs, there is a dramatically higher proportion of stiff RBCs that could not pass through IESs, while the *M*-Chip results reveal a much higher rate of RBC adhesion to macrophages for SS-DeOxy RBCs than that for AA RBCs. In addition, compared with SS-Oxy RBCs, the normalized internalization speed during phagocytosis for SS-DeOxy RBCs can also be much faster. However, most of these sickled SS RBCs require a long time to be digested after engulfment by macrophages, which would eventually slow down the overall elimination speed under hypoxia. In this case, there is an estimated two orders of magnitude higher retention rate $V^{R}$ especially due to the abundant, highly stiffened and much more adhesive-sickled SS RBCs under hypoxia. Such superfast splenic retention rate under hypoxia is expected to overwhelm the RBC elimination capacity with the retention rate becoming much faster than elimination rate ($V^{E}\gg V^{R}$). Consequently, it could result in significant splenic RBC sequestration (Fig. 3*J*). In the case of ASSC, the IESs are quickly blocked and then incoming blood through the open circulation is trapped in the red pulp. This could quickly result in a decrease of ≥2 g/dL in Hb level in circulating blood, and rapid increase in spleen size potentially causing a life-threatening crisis (51). It is important to note that due to the strong heterogeneity of SS RBCs within each SCD patient and due to the differences in hematocrit, HbS, and HbA contents across different patients, significant patient-specific variations are expected with respect to the degree of hypoxia as well as the extent of RBC sequestration in the spleen and the resultant clinical outcome.

### Possible Influence of Endothelial Cell Stiffness.

Previous studies showed that the effective stiffness of endothelial cells is two orders of magnitude (100 times) higher than that of healthy RBCs (52). Our results in Fig. 2 *B* and *D* show that the stiff sickled SS RBCs are about 14 times stiffer than the healthy AA RBCs, therefore the effective stiffness of endothelial cells is estimated to be about seven times stiffer than sickled SS RBCs. We thus assumed that the IES wall composed of endothelial cells is rigid and non-deformable for studying the passage of RBCs through IESs. Nevertheless, it would be interesting to find out how a deformable IES wall would influence the behavior of splenic IES filtration of RBCs in future studies.

### Elimination of SS RBCs by the Splenic Macrophages.

Our *M*-Chip study suggests that splenic macrophages process SS RBCs and AA RBCs in different ways. Under a static condition, as in most of existing phagocytosis assays, phagocytes play an active role. Here the rate of engulfment greatly depends on the physical distance between phagocytes and surrounding targets as well as signaling to activate the internalization machinery. However, erythrophagocytosis in splenic cords takes place under flow. The slower flow velocity and higher potential for cell adherence in the splenic cords provide greater opportunity and time for SS RBCs to interact with macrophages compared to AA RBCs.

Our results have shown that SS RBCs are prone to retention by the macrophages under a slow blood flow compared to AA RBCs, and this phenomenon is significantly enhanced by hypoxia. Such higher retention would greatly shorten the spatial and temporal sensing and binding steps involving the phagocytic receptors. This in turn would likely enhance the engulfment process of SS RBCs in the spleen. Moreover, sickled RBCs under hypoxia show expedited engulfment by macrophages compared to the case of non-sickled RBCs either under normoxic or hypoxic conditions. These phenomena are possibly associated with multiple biochemical mechanisms, including hypoxia-enhanced PS exposure (53, 54), Band 3 clustering (26, 55–59), and CD47 expression (25, 60–62) on SS RBC membrane, thereby facilitating the recognition and engulfment of sickle cells by macrophages (59, 63, 64). In addition to the interfacial biochemical factors, our study shows that sickled SS RBCs exhibit altered biophysical properties (abnormal shapes and increased rigidity) as opposed to AA RBCs and non-sickled SS RBCs, which may also play the analogous role in promoting the elimination of RBCs by the macrophages (28, 65–67). These findings suggest that, in addition to concurrent intravascular hemolysis due to RBC fragility, the accelerated retention and elimination of SS RBCs by the splenic macrophages (i.e., extravascular hemolysis) may also contribute to the shortened lifespan of RBCs and hemolytic anemia in SCD.

### Reoxygenation by Blood Transfusion Can Contribute to the Regression of ASSC-Induced Splenomegaly.

Urgent management of ASSCs relies on the immediate transfusion of normal AA RBCs (20). In addition, some clinical practice recommends lower-dose blood transfusion for ASSC patients in order to avoid the risks of high post-transfusion hemoglobin values due to the anticipated quick release of sequestrated blood from spleen (68). We hypothesize that rapid reoxygenation provided by the transfusion-induced inflow of deformable oxygenated normal AA RBCs restores an efficient circulation of RBC in the spleen and consequently allows for the retained sickled SS RBCs to enter back into circulation. Our results validate this premise and indeed show that reoxygenation rapidly resumes the ability of the sickled SS RBCs originally retained by the micro-slits to cross the slits in our *S*-Chip module. It also promotes the fusion/digestion of phagocytosed sickled SS RBCs in the macrophages in our *M*-Chip module. Both processes contribute directly to reduce the splenic congestion which in turn alleviate congestion-induced hypoxia and release of the retained SS RBCs in the spleen.

### Future Perspectives.

Our approach explores intrasplenic cellular processes that cannot be observed directly in human subjects, at least dynamically. Fig. 2 *F*–*J* show that observations in the *S*-Chip closely match static histological observations in human spleens either from “spleen-healthy” subjects or from patients with SCD, confirming that our experimental setup provides a close mimicry of the complex splenic retention/accumulation process of RBCs in SCD. The proportions of sickled SS RBCs have been shown to be high in the spleen of patients splenectomized for ASSC (17), but no precise quantification has been performed yet. Quantitative extrapolation of our results to the in vivo situation will require such quantification, the interpretation of which will be compounded by the fact that the static snapshot of histology reflects the superimposition of processes that occurred during hours/days before splenectomy. The accelerated blockage in the micro-slits of heated RBCs observed in vitro (Fig. 2*K*) also matches their rapid clearance observed in vivo in humans with accumulation in the spleen (69). The results presented here thus provide a framework for a relevant quantitative interpretation of future histological and RBC clearance studies in human subjects.

In summary, our oxygen-regulated spleen-on-a-chip (including *S*-Chip and *M*-Chip) provides an in vitro platform to investigate how the spleen maintains a homeostatic balance between retention and post-retention processing of altered RBCs. Disruption of this balance leads to splenomegaly because retained RBCs, left undestroyed, accumulate in the organ causing it to swell. This culminates in acute splenic sequestration in SCD. Our integrated slits-and-macrophages microfluidic platform also has the potential to enable mechanistic exploration of the pathophysiology of other hemolytic disorders (14, 70–72). Splenomegaly and anemia are hallmarks of major inherited and acquired RBC diseases such as hereditary spherocytosis and malaria, amongst others. Hereditary spherocytosis is a natural model of mechanical retention of abnormally shaped RBCs in the spleen (73, 74). In this disease, splenectomy is a powerful cure for anemia in severe cases. In malaria, splenomegaly and anemia are tightly associated (75), and retention is an important mechanism of natural protection and parasite survival, as recently confirmed by a splenectomy study in Indonesia (47, 76). Splenic retention and macrophage-mediated processing of RBCs also impact transfusion efficacy (77). Many protective or pathogenic mechanisms are still not fully understood in these diseases. The combination of our *S*-Chip and *M*-Chip, provides a potential pathway to elucidate the mechanisms underlying the functions of the human spleen in physiology and disease with possible applications in disease diagnosis, patient treatment, and drug efficacy assessments.

## Materials and Methods

### Experimental Setup.

The microfluidic devices used in our *S*-Chip module and *M*-Chip module were fabricated following standard fabrication processes. More details are given in **SI Appendix*, *Experimental Setup for In Vitro Experiments**.

### Sample Preparation.

Healthy blood samples were obtained from the local blood bank. Sickle blood samples were drawn from homozygous SCD patients at the Massachusetts General Hospital under an Excess Human Material Protocol approved by the Partners Healthcare Institutional Review Board (IRB) with a waiver of consent. Additional HbSS blood samples were drawn from SCD patients at the University of Pittsburgh under University of Pittsburgh IRB protocol PRO08110422. In vitro microfluidic experiments were conducted under an approved exempt protocol (Massachusetts Institute of Technology IRB protocol E-1523). THP-1 macrophages were cultured in the cell channel of the microfluidic device. More details are given in **SI Appendix*, *Sample Preparation for In Vitro Experiments**.

### Statistical Study.

Data were analyzed using GraphPad software (GraphPad Software). All data were expressed in terms of statistical mean ± SD, except stated otherwise. A two-sample *t* test was used to generate the *P* values between measurements for different cell populations. A one-way ANOVA test was used to generate *P* values for comparison of more than two groups. *P ≤ 0.05 was considered statistically significant.

## Supplementary Material

Appendix 01 (PDF)

Movie S1. Mechanical filtration of AA RBCs under normoxia by micro-slits in the S-chip. (Movie is sped up 4×)

Movie S2. Mechanical filtration of SS RBCs under normoxia by micro-slits in the S-chip. (Movie is sped up 4×)

Movie S3. Faster mechanical retention of SS RBCs under hypoxia by micro-slits in the S-chip. (Movie is sped up 4×)

Movie S4. Sickled SS RBCs, originally blocking all micro-slits under hypoxia, unsickle upon reoxygenation, resulting in the unblocking of those originally blocked slits. (Movie is sped up 4×)

Movie S5. Macrophage-retention process of SS RBCs being progressively adhered on the surface of an individual macrophage under hypoxia.

Movie S6. A sickled SS RBC under hypoxia strongly attached to a macrophage with its sharp spicules under the flow.

Movie S7. A representative phagocytosis process of a non-sickled SS RBCs with a regular biconcave disc shape under normoxia. (Movie is sped up 64×)

Movie S8. A representative phagocytosis process of an irreversibly sickled SS RBC under normoxia. (Movie is sped up 64×)

Movie S9. A representative prolonged phagocytosis process of a SS RBC under normoxia. (Movie is sped up 64×)

Movie S10. A representative phagocytosis process of non-sickled SS RBCs under hypoxia. (Movie is sped up 64×)

Movie S11. A representative phagocytosis process of sickled SS RBCs (type I) under hypoxia. (Movie is sped up 64×)

Movie S12. A representative phagocytosis process of heated AA RBCs under hypoxia. (Movie is sped up 64×)

Movie S13. A representative phagocytosis process of sickled SS RBCs (type II) showing prolonged digestion process under hypoxia. (Movie is sped up 64×)

Movie S14. Phagocytosis of an SS RBC during a successive deoxygenation and reoxygenation process. (Movie is sped up 64×)

Movie S15. An internalized sickled SS RBC showed obvious expedited fragmentation process upon the reoxygenation. (Movie is sped up 64×)

Movie S16. Comparison of animated motions between “slit-passible” RBCs (left) and “slit-unpassable” RBCs (right) through the IESs in the DPD simulation.

## Data Availability

All study data are included in the article, *SI Appendix*, and Movies S1-S16.
