# Supplementary material for: Microfluidic study of retention and elimination of abnormal red blood cells by human spleen with implications for sickle cell disease
Source: Proc Natl Acad Sci U S A. 2023 Feb 2;120(6):e2217607120. doi: 10.1073/pnas.2217607120 (PMC9963977; doi:10.1073/pnas.2217607120)
Supplement: Supplementary file 1 — Appendix 01 (PDF) [file pnas.2217607120.sapp.pdf]

PNAS
www.pnas.org

Supplementary Information for

Microfluidic study of retention and elimination of abnormal red blood cells by human spleen with implications for sickle cell disease

Yuhao Qiang[a], Abdoulaye Sissoko[b,c,d], Zixiang L. Liu[e], Ting Dong[f], Fuyin Zheng[a,g], Fang Kong[g], John M. Higgins[h], George E. Karniadakis[e], Pierre A. Buffet[b,c,d,1], Subra Suresh[a,i,1], Ming Dao[a,g,1]

[a]Department of Materials Science and Engineering, Massachusetts Institute of Technology, Cambridge, MA 02139
[b]Université Paris Cité, INSERM, Biologie Intégrée du Globule Rouge, 75015 Paris, France
[c]Université des Antilles, Biologie Intégrée du Globule Rouge, 75015 Paris, France
[d]Laboratoire d'Excellence du Globule Rouge, 75015 Paris, France
[e]Division of Applied Mathematics, Brown University, Providence, RI 02912
[f]Koch Institute for Integrative Cancer Research, Massachusetts Institute of Technology, Cambridge, MA 02139
[g]School of Biological Sciences, Nanyang Technological University, 639798 Singapore
[h]Massachusetts General Hospital, Harvard Medical School, Boston, MA 02114
[i]Nanyang Technological University, 639798 Singapore

[1]To whom correspondence may be addressed. Email: ssuresh@mit.edu, pabuffet@gmail.com, or mingdao@mit.edu.

This PDF file includes:

Supplementary text

Figs. S1 to S4

Tables S1 to S3

Captions for Movies S1 to S16

References for SI reference citations

Other supplementary materials for this manuscript include the following:

Movies S1 to S16

Supplementary Information

Experimental setup for *in vitro* experiments

The microfluidic device was fabricated by permanent covalent bonding of polydimethylsiloxane (PDMS) channels and the glass substrate following air plasma treatment for 1 minute in a plasma cleaner (Model PDC-001, Harrick Plasma, Ithaca, NY, USA). The PDMS channels were fabricated by casting customized SU-8/Si master molds with a degassed PDMS mixture of base and curing agent (10:1, w/w) using the soft-lithography technique. The SU-8/Si master molds were fabricated following standard photolithography techniques (1). The double-layer microfluidic devices were made by bonding two microchannels with an in-between layer of oxygen-exchange PDMS membrane (150 μm thick). The cell channel was loaded with RBC suspension by injection with a syringe pump (Harvard Apparatus) or external hydraulic columns via tygon tubing, and the gas channel was connected to gas supplies via tygon tubing at a regulated pressure (4.5 psi). Normoxic and hypoxic conditions of cells were obtained by switching between two gas supplies of different gas mixtures at different oxygen levels, respectively: 20% oxygen (O_2), 5% carbon dioxide (CO_2) with the balance of nitrogen (N_2), and an oxygen poor gas mixture: 2% O_2, 5% CO_2 with the balance of N_2. The blood perfusion and erythrophagocytosis processes were observed and recorded via a high-resolution CMOS camera (The Imaging Source, Charlotte, NC, USA) which was mounted on an Olympus X71 inverted microscope (Olympus America, Breinigsville, PA, USA) with a 60x oil-immersion objective (NA=1.25). Time-lapse imaging of adhesion and erythrophagocytosis processes in *M*-Chip was performed under a standard condition (5% CO_2, 37 °C, 95% relative humidity) using a stage top incubation system (ibidi, Gräfelfing, Bayern, DE). For fluorescent and confocal microscopy, RBCs and THP-1 macrophages were labeled using PKH26 and PKH67 dyes (Sigma-Aldrich, St. Louis, MO, USA), respectively. Then the cells were rinsed by PBS and fixed using 4% Formaldehyde before imaging.

Sample preparation for *in vitro* experiments

Healthy blood samples were obtained from the local blood bank. Sickle blood samples were drawn from homozygous SCD patients at the Massachusetts General Hospital under an Excess Human Material Protocol approved by the Partners Healthcare Institutional Review Board (IRB) with a waiver of consent. Additional HbSS blood samples were drawn from SCD patients at the University of Pittsburgh under University of Pittsburgh IRB protocol PRO08110422. *In vitro* microfluidic experiments were conducted under an approved exempt protocol (Massachusetts Institute of Technology IRB protocol E-1523).

HbSS blood samples used in this study were restricted to patients with no transfusions for at least 30 days prior to blood draw. Blood samples were collected in 4 ml K2 ethylenediaminetetraacetic acid (EDTA) spray-coated anticoagulant vacutainers. Blood samples were shipped to Massachusetts Institute of Technology on melting ice and stored at 4 °C. Hemoglobin fractions were determined with cellulose agar electrophoresis and confirmed by high performance liquid chromatography. All experiments were conducted within 5 days of blood draw. The complete blood count (CBC) and hemoglobin electrophoresis data for all HbSS blood samples are summarized in Table S1. The packed red blood cells (pRBCs) were gently washed twice with phosphate buffered saline (PBS) solution (Sigma-Aldrich, St. Louis, MO, USA) at 2,000 rpm for 2 min at room temperature (20 °C). The hematocrit was measured using the ZipCombo centrifuge (LW Scientific, Lawrenceville, GA, USA). The pRBC pellet was resuspended in PBS with 1% (w/v) bovine serum albumin (BSA) (EMD Millipore, Billerica, MA) at a desired concentration. pRBC suspensions were stored at 4 °C until use. The heated RBCs were prepared by incubating a vial

of packed AA RBCs in a water bath at 50 °C for 15 minutes. For RBC opsonization, pRBCs were suspended in PBS (2%, v/v), and incubated with ~ 0.5 μM IgG (Rockland, Limerick, PA, USA) at room temperature (20 °C). Cell line THP-1 cells (ATCC, Manassas, VA, USA) were cultured in complete RPMI media (StemCell Technologies, Cambridge, MA, USA) supplemented with 10% fetal bovine serum (FBS) (Sigma-Aldrich, St. Louis, MO, USA). THP-1 cells were differentiated using 100 ng/ml phorbol myristate acetate (PMA) (StemCell Technologies, Cambridge, MA, USA) for 2 days and confirmed by cell attachment to the substrate in the microfluidic channel.

Ex vivo experiments on human spleens

Human spleens used for the *ex-vivo* experiments were collected through Spleenvivo project, a protocol approved by the "Ile-de-France II" IRB on 4 September 2017 (#CPP 2015-02-05 SM2 DC) (2). Details for the *ex vivo* experiments of the three cases corresponding to the descriptions in Fig.2 *H*-*J* are as follows:

Case I Normal spleen + AA RBC "RHNP1" (Fig. 2*H*): this spleen was retrieved from a 53-year-old female patient (without an underlying RBC-related disease) who underwent a splenopancreatectomy for pancreas cancer.
Case II Normal spleen + SS RBC "RH98" (Fig. 2*I*): this spleen was retrieved from a 75-year-old female patient who underwent splenopancreatectomy for pancreatic cancer (adenocarcinoma but with no RBC-related disease). Her spleen was collected through the Spleenvivo protocol. This spleen was perfused *ex vivo* as described in a prior study (3), with a mixture of AA-RBC and SS-RBC from a SCD adult patient benefiting from transfusion (HbS = 26% post-exchange transfusion) from a study entitled "Pathophysiological Exploration of Red Blood Cells" (https://clinicaltrials.gov/ct2/show/NCT03541525?term=NCT03541525).
Case III SS RBC in an acute splenic sequestration crisis (ASSC) spleen "RSS5" (Fig. 2*J*): this spleen was retrieved from an eight-year-old male child living with SCD who underwent a partial splenectomy for hypersplenism. This child had a history of vaso-occlusive crises and ASSC. He was treated with hydroxyurea (20 mg/kg/day). He was transfused with AA-RBC at 12 days and 2 days (HbS = 44% post-exchange transfusion) prior to splenectomy in preparation for the partial splenectomy.
All the spleens for the *ex-vivo* experiments were retrieved and sections were prepared immediately after surgery (**Case I** and **Case III**) or post-perfusion (**Case II**), processed, Giemsa-stained and histological analysis were carried out as described in earlier work (4).

Modelling of mechanical filtration of RBCs by micro-slits and IESs

As a red blood cell (RBC) is forced to traverse the constriction of a relatively long micro-slit under a constant pressure difference (ΔP), it must reach a maximum value of its major axis in the slit (L_s) (5). We can then estimate the critical shear modulus (μ_C) for RBCs to pass through the slits using the Young–Laplace equation (6, 7) with a geometrical adjustment factor f_g:

$$\frac{(\Delta P \times r_a)}{f_g \mu_C} = 2.45\frac{L_s}{r_a}, \quad \left(\frac{L_s}{r_a} > 1\right) \tag{S1}$$

where r_a denotes the minor radius of the RBC. Given that the RBC has a constant volume (~ 95 μm^3) and its minor axis equals to the width of slit (3 μm) while it squeezes through the slit, we estimate the maximum extension length of the RBC (L_s) to be ~ 6.3 μm and the minor radius (r_a) to be ~1.5 μm (Fig. S1*A*). As the first order approximation, $f_g = 1$ is taken in this study for estimating the critical shear modulus μ_C. In our experiments, the pressure difference (ΔP) across the slits is estimated to be ~100 Pa corresponding to an upstream RBC fluid velocity of 100 μm/s

using the simulation software COMSOL Multiphysics 5.2 (COMSOL, Inc., Burlington, MA, USA) shown in Fig. S1*B*. Using the above-mentioned parameters in Eq. (S1) for our *S*-Chip experiments, we estimate the critical shear modulus (μ_C) to be ~ 14.6 μN/m. As shown in Fig. S1*C* and S1*D*, we also performed a systematic estimation of the probability of a suspended single RBC to either pass through or to be retained while flowing through a micro-slit of different geometrical dimensions. For this purpose, we invoked an upstream fluid velocity of 100 μm/s using the Dissipative Particle Dynamics (DPD) computational simulation model, details of which have been reported in our earlier work (8). The critical shear modulus of the RBC was estimated to vary from 4.1 to 22.3 μN/m at the velocity of 100 μm/s (see Movie S16). The critical shear modulus $\mu_C \sim 14.6\ \mu\mathrm{N/m}$, estimated previously using Eq. (1), agrees well with the DPD results using the same slit geometry as used in experiments. In particular, through the DPD simulations, we found that the critical shear modulus for our *in-vitro* *S*-Chip design (which features rectangular slits 15 μm in length and 3 μm in gap width) has geometrical equivalence to that of the slits with a circular or square cross-section but a narrower gap width (*d* = 1.8~2.0 μm) (Fig. S1*D*), where the geometry of the latter is closer to that of a narrow IES in the human spleen (9).

On the basis of the foregoing considerations, we set the critical shear modulus of RBCs as 14.6 μN/m. In other words, RBCs with membrane shear modulus larger than this value, μ_C>14.6 μN/m, will not be expected to pass the slits in our *S*-chip. We could thereby estimate the fraction of "slit-unpassable" RBCs ($F = \frac{N_{\mu>\mu_C}}{N_{total}}$) from the population of different types of RBCs. The values of *F* were estimated in the present study to be 0.6% for both AA-Oxy RBCs and AA-DeOxy RBCs, 6.1% for Heated: AA=1:4 RBCs, 20.6% for SS-Oxy RBCs, and 64.3% for SS-DeOxy RBCs, respectively, based on the measured shear modulus values shown in Fig. 2*B*.

During the perfusion process involving the micro-slits in the *S*-chip, we have found that the number of blocked slits by those "slit-unpassable" RBCs increases with time. To correlate the mechanical filtration behavior of RBCs with the fraction of "slit-unpassable" RBCs (*F*) in different samples, we have developed a simple RBC-filtration model inspired by a particle-filtration model named "Complete Blocking Model" (10), allowing for the approximation of the proportion of slits remained open (P_{Open}) as a function of time (*t*) by the expression:

$$P_{\mathrm{Open}} = 1 - \sum_{i=1,n} P_i \tag{S2}$$

where $P_i = \frac{N_i}{N_S}$ *is* the added proportion of blocked slits at each filtration step, N_S is the initial number of open slits ($N_S = 32$ for our *S*-Chip, see Fig. S1*E*), N_i is the number of additional blocked slits at each filtration step estimated as $(N_S - \sum_{j=0,i-1} N_j)\frac{(N_S - \sum_{j=0,i-1} N_j)}{N_C} F$ (for $i = 1, \ldots, n$), i is the step number in a continuous filtration process, n is the total accumulated steps of filtration with time ($= \frac{t}{t_c}$), and $N_0 = 0$. t_c is the characteristic transit time of individual RBCs passing through the slit, which is assumed to be a constant ($t_c = 0.1s$). N_C is the cell number in the vicinity of the slits of each step, which is assumed to be a constant (N_c is taken to be 600 for our *S*-Chip). $(N_S - \sum_{j=1,\ldots,i-1} N_j)$ is the number of slits remained open at the beginning of each current filtration step. $\frac{(N_S - \sum_{j=1,\ldots,i-1} N_j)}{N_C} F$ is the probability of blockage by the slits remained open at each current filtration step. The numerical simulation was performed using MATLAB (MathWorks, Natick, MA, USA). Fig.S1 *F* provides our simulation results of the estimated proportion of slits that remained open as a function of time for different RBC subpopulations.

Retention of RBCs at splenic slits under mild and severe hypoxia

The spleen is in general under some degree of hypoxia, which ranges from a mild hypoxic condition to a severe hypoxic condition (1-13%) depending on the location (see Table S2). Using our sickling kinetics assay, we found that the sickling rate and sickled fraction are much lower under 5% O_2 (mild hypoxia) than those under 2% O_2 (severe hypoxia) for the same individual SS RBCs from three SCD patients (n=3) (Fig. S3*A*). As discussed in the main text, RBC retention at IES is highly dependent on the transient sickled fraction of SS RBCs when they pass through the slits. For instance, at t = 40s, only 17% SS RBCs were sickled under 5% O_2, while 82% RBCs were sickled under 2% O_2 (Fig. S3*B*). Additionally, in our *S*-chip filtration experiments, we didn't see significantly different mechanical retention responses between SS RBCs under 5% O_2 (n=6) and those under normoxia (20% O_2) (n=11) in contrast to the severe hypoxic condition (2% O_2) (n=8) (Fig. S3*C*). In other words, the homeostatic balance could still be maintained in mild hypoxia (~5%) in the spleen, which could help explain why ASSC doesn't occur continually and only a small portion of children (5 ~ 10%) suffer ASSC. However, our results show that SS RBCs still generally show a higher propensity for splenic retention compared to AA RBCs even under a normoxic condition. This progressively impedes blood flow and induces lower oxygen level in the spleen, and further leads to higher risk of sickling of SS RBCs while they traverse the slits. Therefore, ASSC could be considered progressive deterioration process due to a gradually deepening hypoxic condition in the spleen. However, once the severe hypoxia is achieved, severe splenic sequestration could occur rapidly.

Mechanical filtration of *P. falciparum*-invaded RBCs by micro-slits

Splenic retention of RBCs arising from their stiffening is not unique to SCD, but can also occur in other diseases such as *P. falciparum* malaria where parasitized RBCs can be significantly stiffened *in vivo*. Our *S*-chip module can also be employed to model the mechanical filtration behavior of human RBCs invaded by P. falciparum (*Pf*-RBCs) through the splenic IESs. *Pf*-RBCs were found to show decreased cellular volume and deformability in different intraerythrocytic stages from uninfected to ring stage (early stage after the invasion by the parasites) and trophozoite stage (later stage as the parasite undergo morphological changes to mature trophozoites) (11-14). Fig. S4 shows our predicted results of comparing healthy RBCs, ring stage *Pf*-RBCs at various parasitemia levels and trophozoite stage *Pf* -RBCs based on their volume and associated shear modulus data measured by diffraction phase microscopy (DPM) in the previous study (15). The values of L_s and μ_C for the ring *Pf* -RBCs and trophozoite *Pf*-RBCs were estimated based on their cell volume and the slit dimensions of our *S*-Chip, respectively (see Table S3). The fractions of "slit-unpassable" RBCs (*F*) were estimated to be 0.6% for healthy (0% parasitemia) RBCs, 1.2% for 2% parasitemia ring *Pf*-RBCs, 6.7% for 20% parasitemia ring *Pf*-RBCs, 15.8% for 50% parasitemia ring *Pf* -RBCs, and 86% for 100% parasitemia trophozoite *Pf*-RBCs, respectively. Fig. S4*A* shows the predictions of the proportion of slits remained open as a function of time for *Pf*-RBCs at various parasitemia levels from 0%, 2%, 20%, 50% to 100% at the ring stage, and 100% parasitemia at the trophozoite stage, respectively. The modeling results show the apparent increase of retention rate of *Pf*-RBCs by the IESs with the parasitemia levels as well as the infection stages. We also compared the mechanical retention of 20% parasitemia ring *Pf*-RBCs at physiological temperature (37°C) with those at febrile temperature (41°C). The values of *F* were estimated to be 6.7% and 15.1%, respectively. The *Pf*-RBCs show greater retention rate at higher temperature (Fig. S4*B*).

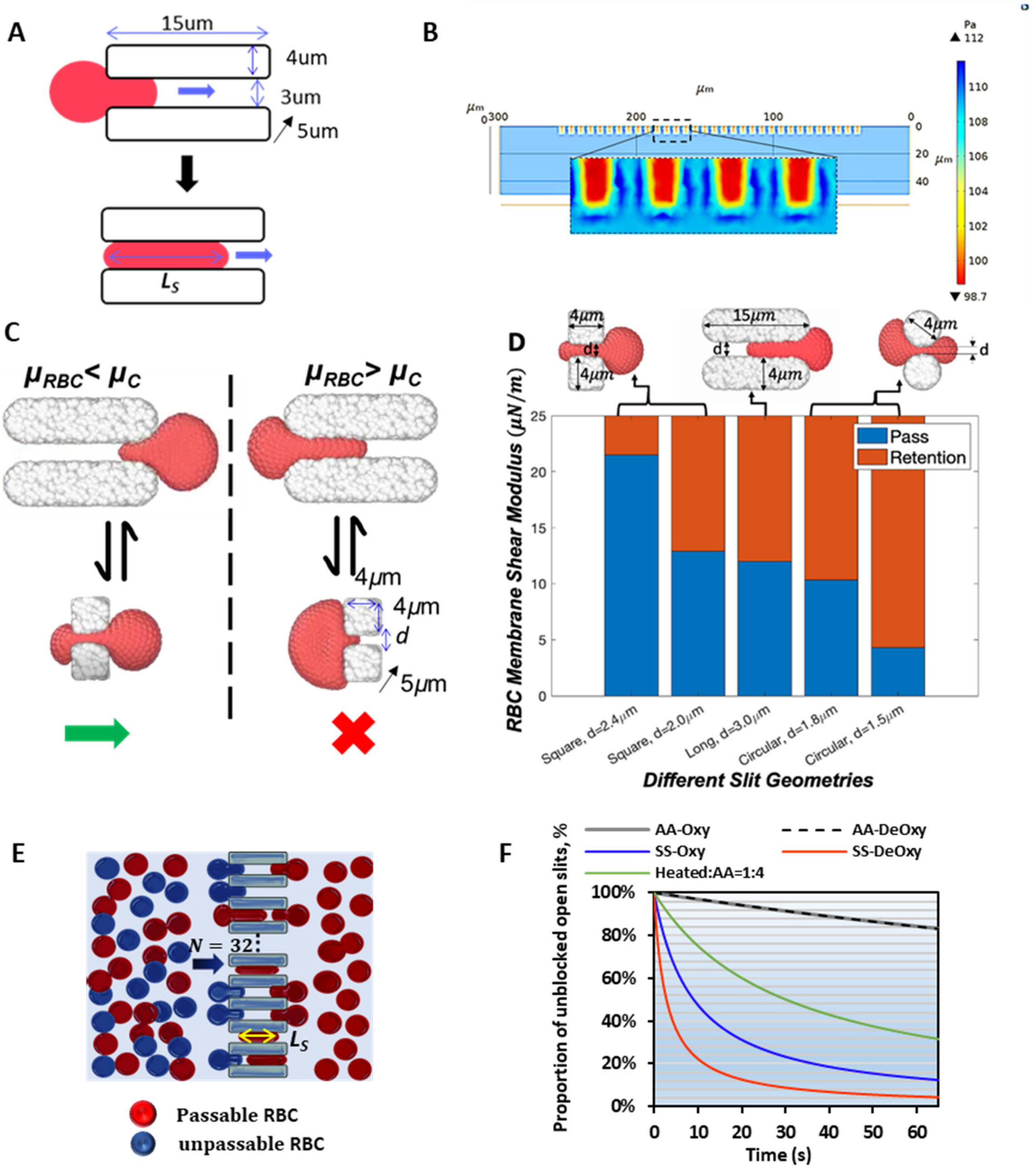

Fig. S1. Estimation of critical shear modulus of slit-passible RBC through the IES. (A) Schematic of the limiting geometry considered in the theoretical modeling. The upper panel shows an RBC entering the slit. The lower panel shows an RBC reaching a maximum value of its major axis during its passage through the slit. (B) Pressure contours of flow through the *S*-chip simulated using the COMSOL computational modeling package. (C) DPD simulation of RBC passage/retention at the slits with two geometrically different but mechanically similar designs. (D) The similitude and difference in terms of RBC shear modulus for RBC passage or blockage at the slits with different geometrical features. Slits with a square and circular cross-section are selected in comparison with the "long" used for the current *in vitro S*-chip design. (E) Schematic illustration of the filtration process of RBCs through the micro-slits. Blue arrows denote the flow direction. (F) Estimation of mechanical retention of different RBC samples/subpopulations in the experiments using the simple RBC-filtration model.

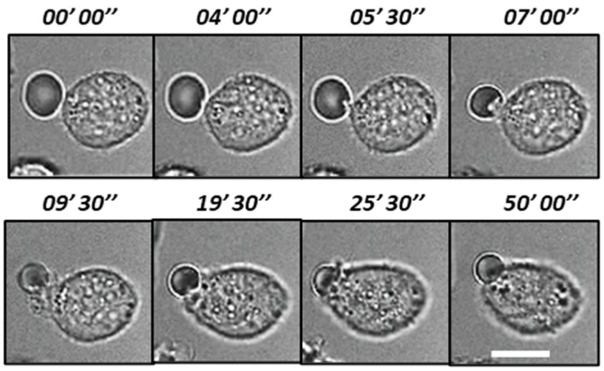

Fig. S2. Time-lapse imaging shows a prolonged phagocytosis process of a non-sickled SS RBC under normoxia (also see Movie S9). Scale bar represents 10 μm.

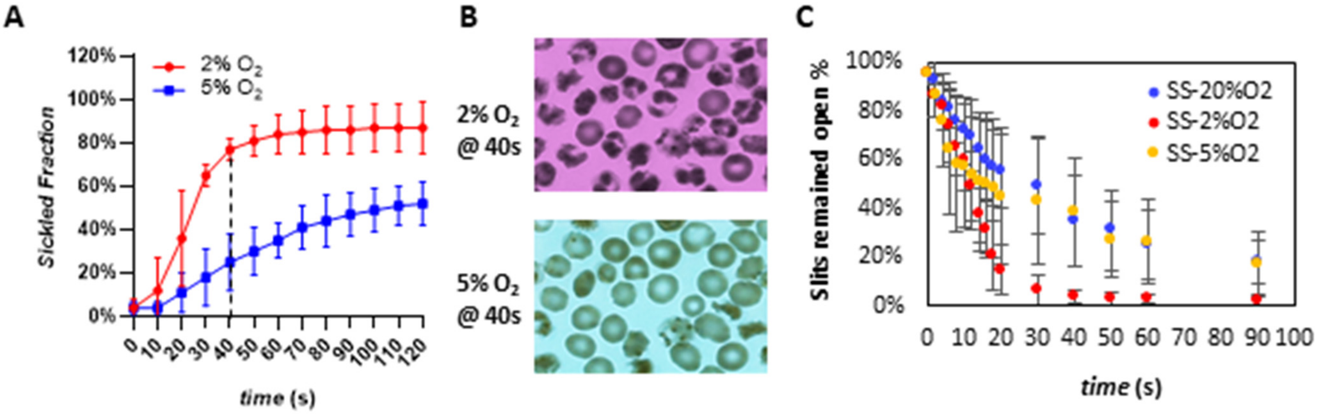

Fig. S3. SS RBCs show faster sickling and micro-slit retention under 2% O_2 than under 5% O2. (A) Comparison of sickling kinetics between two different levels of hypoxia (5% O_2 *vs* 2% O_2) for an identical population of SS RBCs. (B) Representative microscopic images of SS RBCs after deoxygenation for 40 s under 2% O_2 and 5% O_2, respectively. (C) Comparison of mechanical retention behaviors of SS RBCs under 20% O_2, 2% O_2 and 5% O_2, respectively.

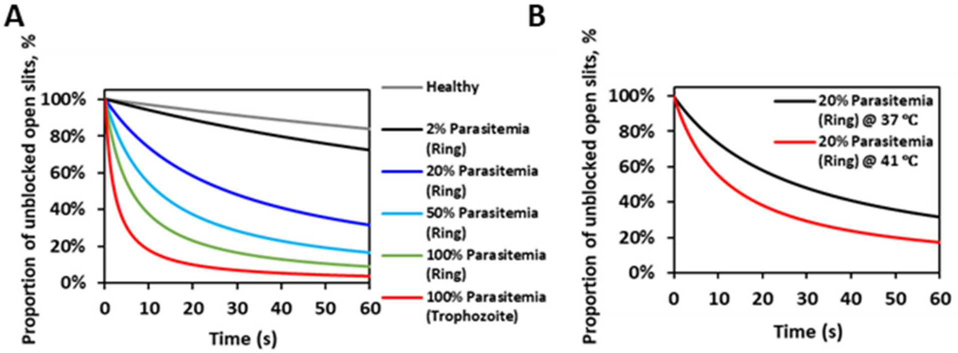

Fig. S4. Prediction of mechanical retention of Plasmodium falciparum infected RBCs (*Pf* -RBCs). (A) Prediction of mechanical retention of *Pf*-RBCs at various parasitemia levels from 0%, 2%, 20%, 50% to 100% at the ring stage, and 100% parasitemia at the trophozoite stage, respectively. (B) Predictions for mechanical retention of 20% parasitemia Ring *Pf*-RBCs at the different temperatures of 37°C and 41°C, respectively. See *SI Appendix, Modelling of mechanical filtration of RBCs by micro-slits and IESs* for details.

Table S1. Clinical Measurements of sickle cell blood samples. Complete blood count (CBC) data: the white blood count (WBC), hematocrit (HCT), mean corpuscular volume (MCV) and mean corpuscular hemoglobin concentration (MCHC). Hemoglobin electrophoresis data: the fraction (%) of hemoglobin S (HbS), hemoglobin F (HbF), hemoglobin A (HbA), and hemoglobin A2 (HbA2).

	Genotype	WBC	HCT	MCV, fl	MCHC, g/dl	HU	HbS	HbF	HbA	HbA2
**Patient 01**	HbSS	12.13	23.3	69.8	35.2	N	93.7%	2.6%	0.0%	3.7%
**Patient 02**	HbSS	16.02	24.2	101.7	33.5	N	86.1%	11.3%	0.0%	2.6%
**Patient 03**	HbSS	10.03	17.2	72	34.3	N	89.1%	7.4%	0.0%	3.5%
**Patient 04**	HbSS	15.36	25	87.4	33.2	Y	88.3%	8.7%	0.0%	3.0%
**Patient 05**	HbSS	12.42	27.9	88	35.5	Y	67.7%	29.8%	0.0%	2.5%
**Patient 06**	HbSS	12.01	25.5	90.7	32.9	Y	88.5%	8.5%	0.0%	3.0%
**Patient 07**	HbSS	12.8	19.6	85.8	34.6	N	78.9%	7.1%	0.0%	4.2%
**Patient 08**	HbSS	5.3	29.8	120.7	35	Y	50.3%	31.6%	0.0%	1.7%
**Patient 09**	HbSS	13.7	23.9	99.5	33.6	Y	77.6%	8.1%	0.0%	4.8%
**Patient 10**	HbSS	12.95	17.1	100.0	33.3	Y	83.2%	14.5%	0.0%	2.3%
**Patient 11**	HbSS	6.13	27	84.4	35.9	Y	50.2%	2.3%	0.0%	4.2%
**Patient 12**	HbSS	17.67	24.9	83	34.9	Y	81.5%	15.2%	0.0%	3.3%
**Patient 13**	HbSS	6.63	23.6	71.1	35.2	N/A	50.0%	5.2%	0.0%	3.4%
**Patient 14**	HbSS	5.33	23.4	84.8	35.9	Y	49.8%	2.6%	0.0%	4.0%
**Patient 15**	HbSS	19.91	23	83	35.2	Y	85.9%	10.7%	0.0%	3.4%
**Patient 16**	HbSS	7.75	25.3	86.9	34.8	Y	49.9%	3.1%	0.0%	3.5%
**Patient 17**	HbSS	9.49	36.4	71.9	36.5	N/A	50.9%	1.3%	0.0%	4.4%
**Patient 18**	HbSS	12.9	22.3	83.2	34.7	N	72.4%	2.2%	10.0%	5.1%
**Patient 19**	HbSS	9.34	23.6	114	33.1	Y	69.0%	25.3%	3.7%	2.0%
**Patient 20**	HbSS	9.71	23.4	70.9	32.9	N/A	70.9%	6.0%	18.7%	4.4%
**Patient 21**	HbSS	5.1	37.7	78.5	36.4	N	62.5%	35.4%	0.0%	2.1%
**Patient 22**	HbSS	13	19.2	102.9	36.6	Y	>80%	3.0%	N/A	N/A
**Patient 23**	HbSS	6.73	20.7	99.0	34.8	Y	76.3%	20.5%	0.0%	3.2%
**Patient 24**	HbSS	14.95	27.9	71.7	31.5	N/A	71.5%	9.7%	14.5%	4.3%
**Patient 25**	HbSS	8.89	20.4	117.2	36.3	Y	77.6%	20.1%	0.0%	2.3%

Table S2. Splenic oxygen level and transit time/velocity of RBCs in different species. IES: inter-endothelial slit; RM: reticular meshwork.

Oxygen level	Species	Ref.	Transit time/velocity	Species	Ref.
1% ~ 5%	mice	(16)	0.02 ~ 60.5 s (IES)	rat	(17)
1% ~ 4%	mice	(18)	20 s	dog	(19)
10 ± 2.4 %	rat	(20)	> 66 s	human	(21)
5 ± 1 %	rat	(22)	7 μm/s, 700 μm/s (IES)	human	(23)
~7%	mice	(24)	130.2 ± 19.2 μm/s (RM)	mice	(25)
3% ~ 13%	rabbit	(26)			

Table S3. Parameters used in the prediction of mechanical retention of *Pf*-RBCs.

	V (μm^3)	L_s (µm)	μ_c (µN/m)
**Healthy RBCs**	94	6.3	14.6
**Ring *Pf* -RBCs**	90	6	15.3
**Trophozoite *Pf* -RBCs**	53.6	3.6	25.5

List of Supplementary Movies

Movie S1. Mechanical filtration of AA RBCs under normoxia by micro-slits in the *S*-chip. (Movie is sped up 4x)

Movie S2. Mechanical filtration of SS RBCs under normoxia by micro-slits in the *S*-chip. (Movie is sped up 4x)

Movie S3. Faster mechanical retention of SS RBCs under hypoxia by micro-slits in the *S*-chip. (Movie is sped up 4x)

Movie S4. Sickled SS RBCs, originally blocking all micro-slits under hypoxia, unsickle upon reoxygenation, resulting in the unblocking of those originally blocked slits. (Movie is sped up 4x)

Movie S5 Macrophage-retention process of SS RBCs being progressively adhered on the surface of an individual macrophage under hypoxia.

Movie S6. A sickled SS RBC under hypoxia strongly attached to a macrophage with its sharp spicules under the flow.

Movie S7. A representative phagocytosis process of a non-sickled SS RBCs with a regular biconcave disc shape under normoxia. (Movie is sped up 64x)

Movie S8. A representative phagocytosis process of an irreversibly sickled SS RBC under normoxia. (Movie is sped up 64x)

Movie S9. A representative prolonged phagocytosis process of a SS RBC under normoxia. (Movie is sped up 64x)

Movie S10. A representative phagocytosis process of non-sickled SS RBCs under hypoxia. (Movie is sped up 64x)

Movie S11. A representative phagocytosis process of sickled SS RBCs (type I) under hypoxia. (Movie is sped up 64x)

Movie S12. A representative phagocytosis process of heated AA RBCs under hypoxia. (Movie is sped up 64x)

Movie S13. A representative phagocytosis process of sickled SS RBCs (type II) showing prolonged digestion process under hypoxia. (Movie is sped up 64x)

Movie S14. Phagocytosis of an SS RBC during a successive deoxygenation and reoxygenation process. (Movie is sped up 64x)

Movie S15. An internalized sickled SS RBC showed obvious expedited fragmentation process upon the reoxygenation. (Movie is sped up 64x)

Movie S16. Comparison of animated motions between “slit-passible” RBCs (left) and “slit-unpassable” RBCs (right) through the IESs in the DPD simulation.

References Cited in Supplementary Information

1. Y. Qiang, J. Liu, M. Dao, E. Du, In vitro assay for single-cell characterization of impaired deformability in red blood cells under recurrent episodes of hypoxia. *Lab on a Chip* **21**, 3458-3470 (2021).
2. C. Roussel *et al.*, Rapid clearance of storage-induced microerythrocytes alters transfusion recovery. *Blood* **137**, 2285-2298 (2021).
3. P. A. Buffet *et al.*, Ex vivo perfusion of human spleens maintains clearing and processing functions. *Blood* **107**, 3745-3752 (2006).
4. S. Kho *et al.*, Evaluation of splenic accumulation and colocalization of immature reticulocytes and Plasmodium vivax in asymptomatic malaria: A prospective human splenectomy study. *PLoS Medicine* **18**, e1003632 (2021).
5. W. B. Haines, Studies in the physical properties of soil. V. The hysteresis effect in capillary properties, and the modes of moisture distribution associated therewith. *The Journal of Agricultural Science* **20**, 97-116 (1930).
6. R. M. Hochmuth, Micropipette aspiration of living cells. *Journal of Biomechanics* **33**, 15-22 (2000).
7. S. Chien, K. L. Sung, R. Skalak, S. Usami, A. Tözeren, Theoretical and experimental studies on viscoelastic properties of erythrocyte membrane. *Biophysical Journal* **24**, 463-487 (1978).
8. Z. L. Liu *et al.*, Computational modeling of biomechanics and biorheology of heated red blood cells. *Biophysical Journal* **120**, 4663-4671 (2021).
9. I. V. Pivkin *et al.*, Biomechanics of red blood cells in human spleen and consequences for physiology and disease. *Proceedings of the National Academy of Sciences* **113**, 7804-7809 (2016).
10. M. Sampath, A. Shukla, A. S. Rathore, Modeling of filtration processes—microfiltration and depth filtration for harvest of a therapeutic protein expressed in Pichia pastoris at constant pressure. *Bioengineering* **1**, 260-277 (2014).
11. S. Huang *et al.*, Dynamic deformability of Plasmodium falciparum-infected erythrocytes exposed to artesunate in vitro. *Integrative Biology* **5**, 414-422 (2013).
12. L. H. Bannister, J. M. Hopkins, R. E. Fowler, S. Krishna, G. H. Mitchell, A Brief Illustrated Guide to the Ultrastructure of Plasmodium falciparum Asexual Blood Stages. *Parasitology Today* **16**, 427-433 (2000).
13. S. Suresh *et al.*, Connections between single-cell biomechanics and human disease states: gastrointestinal cancer and malaria. *Acta Biomaterialia* **1**, 15-30 (2005).
14. J. Mills *et al.*, Effect of plasmodial RESA protein on deformability of human red blood cells harboring Plasmodium falciparum. *Proceedings of the National Academy of Sciences* **104**, 9213-9217 (2007).
15. Y. Park *et al.*, Refractive index maps and membrane dynamics of human red blood cells parasitized by Plasmodium falciparum. *Proceedings of the National Academy of Sciences* **105**, 13730-13735 (2008).
16. C. C. Caldwell *et al.*, Differential effects of physiologically relevant hypoxic conditions on T lymphocyte development and effector functions. *The Journal of Immunology* **167**, 6140-6149 (2001).
17. I. MacDonald, D. Ragan, E. Schmidt, A. Groom, Kinetics of red blood cell passage through interendothelial slits into venous sinuses in rat spleen, analyzed by in vivo microscopy. *Microvascular Research* **33**, 118-134 (1987).
18. R. D. Braun, J. L. Lanzen, S. A. Snyder, M. W. Dewhirst, Comparison of tumor and normal tissue oxygen tension measurements using OxyLite or microelectrodes in rodents. *American Journal of Physiology-Heart Circulatory Physiology* **280**, H2533-H2544 (2001).
19. G. R. Cokelet, Dynamics of erythrocyte motion in filtration tests and in vivo flow. *Scandinavian journal of clinical and laboratory investigation. Supplementum* **156**, 77-82 (1981).
20. S. K. Holland, R. P. Kennan, M. M. Schaub, M. J. D'Angelo, J. C. Gore, Imaging oxygen tension in liver and spleen by 19F NMR. *Magnetic Resonance in Medicine* **29**, 446-458 (1993).

21. I. C. MacDonald, E. E. Schmidt, A. C. Groom, The high splenic hematocrit: A rheological consequence of red cell flow through the reticular meshwork. *Microvascular Research* **42**, 60-76 (1991).
22. D. Jamieson, H. Van den Brenk, Electrode size and tissue pO2 measurement in rats exposed to air or high pressure oxygen. *Journal of Applied Physiology* **20**, 514-518 (1965).
23. V. Brousse, P. Buffet, D. Rees, The spleen and sickle cell disease: the sick (led) spleen. *British Journal of Haematology* **166**, 165-176 (2014).
24. A. C. Groom, The Microcirculatory Society Eugene M. Landis award lecture. Microcirculation of the spleen: new concepts, new challenges. *Microvasc Res* **34**, 269-289 (1987).
25. J. H. Jandl, R. H. Aster, C. Forkner, A. Fisher, R. Vilter, Splenic pooling and the pathophysiology of hypersplenism. *Transactions of the American Clinical Climatological Association* **78**, 9 (1967).
26. J. Vanderkooi, M. Erecinska, I. Silver, Oxygen in mammalian tissue: methods of measurement and affinities of various reactions. *American Journal of Physiology-Cell Physiology* **260**, C1131-C1150 (1991).
